# Intraspecific morphological variation and environmental drivers in *Macleania rupestris*: a model-based population classification

**DOI:** 10.3389/fpls.2025.1645659

**Published:** 2025-08-12

**Authors:** Denisse F. Peña, Paulina Villena, Diana Curillo, Carlos A. Jiménez, Eduardo Ordoñez, Oswaldo Jadán

**Affiliations:** ^1^ Grupo de Biotecnología Agropecuaria, Facultad de Ciencias Agropecuarias, Universidad de Cuenca, Cuenca, Ecuador; ^2^ Instituto Nacional de Investigaciones Agropecuarias (INIAP), Gualaceo, Ecuador; ^3^ Grupo de Ecología Forestal, Agroecosistemas y Silvopasturas en Sistemas Ganaderos, Facultad de Ciencias Agropecuarias, Universidad de Cuenca, Cuenca, Ecuador

**Keywords:** Macleania rupestris, intraspecific variation, morphological traits, environmental gradients, cluster analysis, random forest, Andean forest, ecological restoration

## Abstract

**Introduction:**

*Macleania rupestris*, an ecologically and culturally important species of the Ericaceae family, inhabits the montane forests of southern Ecuador and exhibits significant but understudied intraspecific morphological variation. Understanding this variation and its environmental drivers is crucial for effective conservation and restoration planning, particularly in a changing climate.

**Methods:**

We analyzed 15 quantitative traits in 200 individuals from four populations located in the Azuay and Cañar provinces to identify morphological groups and assess their environmental drivers. Hierarchical clustering and Random Forest classification were employed to detect distinct morphological groups. Additionally, generalized linear models were used to evaluate the influence of climatic seasonality and spatial autocorrelation on the most relevant traits.

**Results:**

We identified two distinct morphological groups, primarily differentiated by seed number per fruit, fruit humidity, fruit length and width, and petiole length traits that together explained over 75% of the observed variation. Climatic seasonality and spatial autocorrelation significantly influenced these key traits. Notably, seed number and fruit length responded strongly to variations in temperature and precipitation, while traits such as fruit humidity and petiole length showed moderate sensitivity to environmental gradients. A Random Forest classification model, based on the most relevant traits, achieved 99.5% accuracy, enabling robust assignment of new individuals into morphological groups.

**Discussion:**

Our findings highlight the influence of environmental heterogeneity on intraspecific differentiation in *M. rupestris* and provide evidence for local adaptation along climatic gradients. This study offers a novel framework for trait-based classification and emphasizes the importance of integrating morphological variation and environmental factors into conservation planning. By identifying environmentally driven morphological groups, these results can inform seed sourcing strategies and restoration efforts aimed at enhancing ecosystem resilience in the montane forests of southern Ecuador.

## 1 Introduction

Trait-based ecology provides a powerful framework for understanding plant function, adaptation, and responses to environmental gradients. Functional trait morphological, physiological, or phenological features that impact fitness offer insights into how plants acquire resources, tolerate stress, and interact with their environments (Violle et al., 2007; Masarovičová et al., 2018; Singh et al., 2024). Intraspecific variability in these traits is increasingly recognized as a key component of ecological and evolutionary processes, especially in heterogeneous systems such as tropical montane forests (Gratani, 2014; Bañares de Dios, 2024; Homeier et al., 2021). Trait-based classification of morphologically similar groups can serve as indicators of plasticity and resilience, facilitating the analysis of environmental influences including altitude, temperature, precipitation, and soil characteristics (Pérez-Camacho et al., 2012; Joshi et al., 2024; Malik et al., 2024). Moreover, trait variation contributes to understanding species’ ecological niches, dispersal strategies, and biotic interactions (March-Salas et al., 2021; Zhang et al., 2022). Recognizing this variation at the intraspecific level is especially relevant for underexplored taxa in biodiversity hotspots, where functional traits may reflect subtle adaptive responses to complex environmental pressures.

*Macleania rupestris* (Kunth) A.C. Sm., commonly known as joyapa, is a woody Andean plant found in the tropical montane forests of southern Ecuador. It belongs to the Ericaceae family, which includes several economically and ecologically important species, especially valued for their edible fruits (Tenuta et al., 2019; Luteyn, 2021). Although specific studies on *M. rupestris* are lacking, species within the Ericaceae family exhibit considerable intraspecific variability, manifested in traits such as leaf size, flower morphology, and fruit structure (Malan, 2013; Larrinaga and Guitián, 2016). These traits are essential for understanding plant responses to environmental gradients and predicting their distribution and adaptability (Díaz et al., 1999; Longhi-Wagner et al., 2012; Tsakalos et al., 2019). Trait-based approaches classify plant species based on their capacities for resource acquisition and allocation in tropical montane environments (Ligarreto et al., 2011; Gratani, 2014). Such classification facilitates the analysis of environmental influences, including altitude, temperature, precipitation, and soil characteristics (Fan et al., 2006; Pérez-Camacho et al., 2012; Wellstein et al., 2013; Souza et al., 2018). Additionally, these morphological traits affect dispersal, resource acquisition, and biotic interactions, shaping species’ ecological niches and habitat distributions (Zambrano et al., 2019; Carvalheiro et al., 2021).

*Macleania rupestris* is a native species of southern Ecuador, primarily inhabiting the mountainous regions of the Tropical Montane Forest. Its natural habitat is characterized by well-drained soils and high humidity, where it mainly grows in hard-to-reach areas, often near ravines or on rocky slopes (Veloza et al., 2014). Also, it is commonly found as a pioneer species in landscapes affected by volcanic activity or recent landslides, thriving along the edges of mature forests and in disturbed environments (Kappelle, 2016). These include forest clearings created by logging or road construction, where human influence has significantly shaped the vegetation dynamics (Luteyn, 2021). This plant has significant ecological value as it provides food and shelter to various fauna, including insects and birds (Luteyn, 2021; Ortiz et al., 2023). From an ethnobotanical perspective, *M. rupestris* is used by local communities both as a food source for livestock and for agro-industrial purposes, such as the production of traditional medicines and the construction of homes (Gori et al., 2022). Its multifunctional value makes it a strategic resource in natural resource management (Báez et al., 2010). In this way, *M. rupestris* plays a key role not only in the ecosystem but also in the local economy (Diazgranados et al., 2021).

However, *M. rupestris* is increasingly threatened by deforestation and forest fires, which degrade the quality of tropical montane forests and the vegetation zones it inhabits (Tapia-Armijos et al., 2015; Arias-Sosa et al., 2021). These activities, primarily driven by agricultural expansion and livestock farming, negatively affect joyapa populations, reducing their distribution and genetic diversity (Richter et al., 2009). Habitat loss also has caused fragmentation of ecosystems, limiting the species’ ability to adapt and survive (Haddad et al., 2015). Furthermore, the deficiency of studies on its intraspecific morphological variation hinders a comprehensive understanding of its adaptive potential and resilience to environmental changes. Forest fires, exacerbated by climate change, are also accelerating the loss of native vegetation (Halofsky et al., 2020). This presents a direct threat to *M. rupestris* and other native species of the region (Richter et al., 2009).

Conducting research to understand and classify morphologically related groups is essential for assessing biodiversity, adaptation, and ecological interactions, particularly in regions with limited taxonomic studies. Identifying these groups is essential to establish a baseline that allows for differentiating populations and subsequently studying their genetic, phenotypic, and adaptive variation (Vandepitte et al., 2014). This knowledge will contribute to the conservation of *M. rupestris* by enabling the design of management strategies that account for the morphological and genetic characteristics of each group. Furthermore, such classification helps identify groups that are more vulnerable to environmental threats, supporting more effective conservation actions. Therefore, clear morphological characterization is key to preserving regional biodiversity (Haider, 2011) and lays the foundation for studies on phylogenetic relationships and adaptive mechanisms. Based on this background, this study was developed to answer the following research questions: 1) Can morphological variation among *M. rupestris* populations be used to identify distinct groups? 2) Which environmental variables, including climate and spatial autocorrelation, influence the most important morphological traits? To answer these questions, we aimed to identify distinct morphological groups, determine the traits most relevant to differentiation, and characterize each group based on trait magnitude. We hypothesize that environmental gradients particularly climatic seasonality and dispersal processes shape morphological variation in *M. rupestris*. Furthermore, we developed a predictive model for classifying new individuals into the identified groups, allowing future applications in conservation, restoration, and biodiversity monitoring.

## 2 Materials and methods

### 2.1 Study area

**Figure 1:**
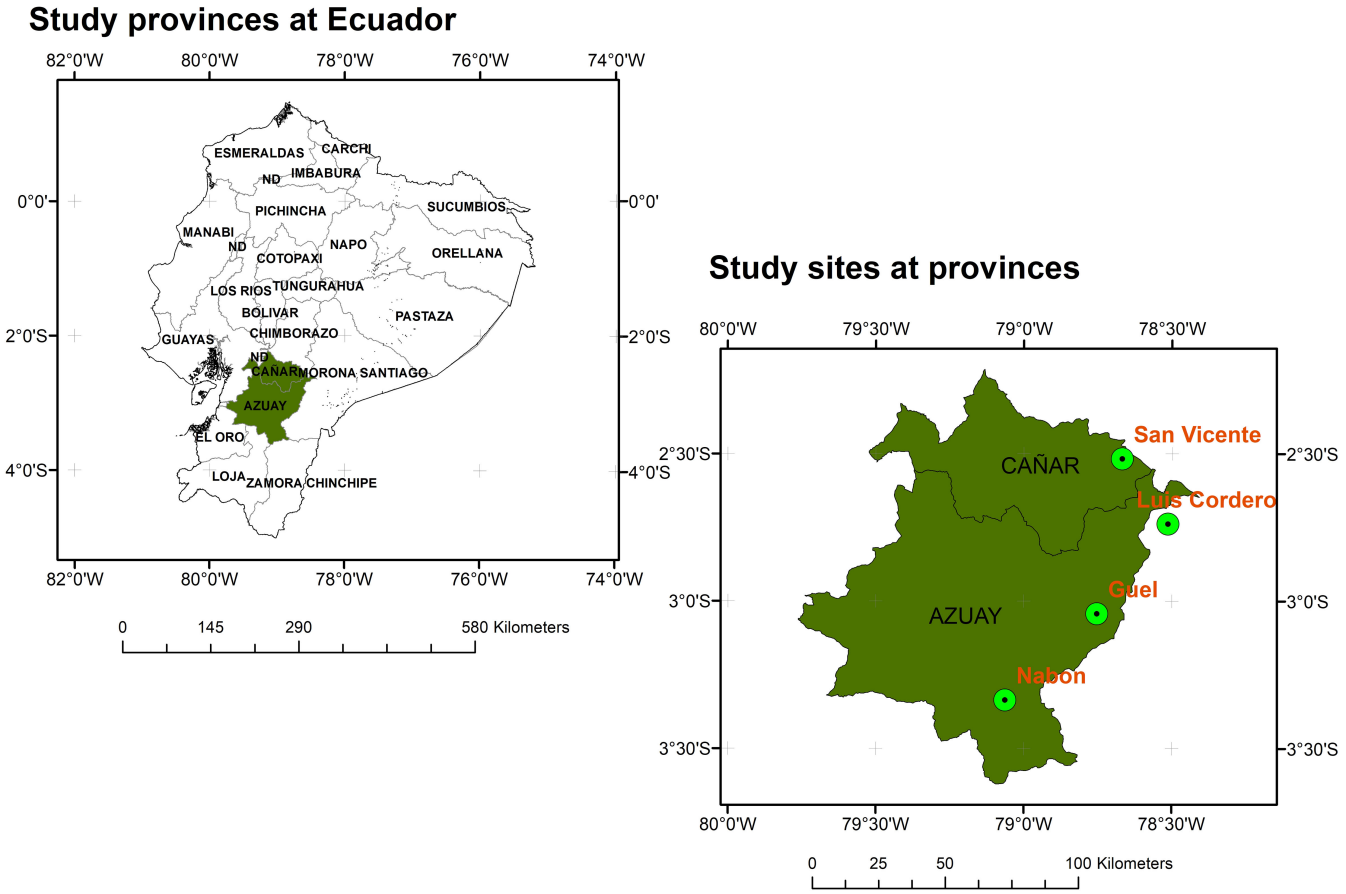
Location of sampling sites for *M. rupestris* individuals across Azuay and Cañar provinces at Ecuador.

The study was conducted in four representative localities within the natural distribution range of *Macleania rupestris* (Ericaceae) in the provinces of Azuay and Cañar, southern Ecuador. The selected sites were Güel (3°03’01.0”N, 78°47’39.6”W) and Nabón (3°20′11.52”N, 79°3′47.14”W) in Azuay province, and San Vicente (2°31’02.0”N, 78°40′02.8”W) and Luis Cordero (2°44’08.4”N, 78°30’48.5”W) in Cañar province (**Figure 1**). These locations are situated in transitional zones between páramo and subpáramo ecosystems, at elevations ranging from 2750 to 3312 m a.s.l. The dominant vegetation includes species such as *Agave sisalana, Baccharis latifolia*, *Miconia calvescens, Racinaea* spp., *Lolium* sp., and *Morella parvifolia.* The regional climate is temperate with an annual average temperature of approximately 11.8 °C in Cañar and 16.3 °C in Azuay. Annual precipitation varies between 800 and 1500 mm depending on the site. Climatic data by site are presented in **Supplementary Table 1** (Fick and Hijmans, 2017). A representative photograph of *M. rupestris* in its natural habitat is provided in **Supplementary Figure 1**, illustrating its shrubby growth form, leaf arrangement, and typical fruit morphology in the montane forest landscape.

### 2.2 Data collection

A total of 200 fertile adult individuals were sampled, with 50 individuals per site. To minimize genetic and environmental redundancy, individuals were spatially separated by a minimum distance. In each locality, 50 reproductive individuals of *Macleania rupestris* were randomly selected, i.e., plants with visible presence of flowers and/or fruits. To reduce the likelihood of sampling branches from the same genotype, a minimum distance of 5 meters was maintained between individuals, considering the shrubby growth habit of the species.

Each plant was characterized using 15 quantitative morphological traits encompassing vegetative and reproductive attributes: petiole length (P, mm), leaf width (LW, cm), leaf length (LEL, cm), rachis length (RL, cm), fruit weight (FW, g), dry fruit weight (DFW, g), fruit length (FL, mm), fruit width (FWD, mm), number of seeds per fruit (SNF), plant height (PH, m), internode length (LL, cm), number of flowers (FNB), number of fruits per branch (FRB), fruit humidity (H, %), and sugar content (BR, °Brix). These traits were selected and adapted from the UPOV (1992) for *Vaccinium* species members of the same botanical family (Ericaceae) and supported by previous studies (Camargo, 1969; Wagstaff et al., 2010; Jin et al., 2010; Carrillo-Perdomo et al., 2015). Morphological data were collected both in the field and laboratory. Plant material was stored in individually labeled plastic bags for transport and identification. Voucher specimens were also collected at each site and deposited at the Azuay Herbarium for taxonomic verification and conservation.

From each plant, four fully developed, mature, healthy, and sun-exposed leaves were collected and analyzed *in situ*, taken from the middle third of the branches. A total of 200 leaves per locality were evaluated. Morphometric variables such as length, width, and petiole length (in cm) were measured, following standardized protocols adapted from (Wilbur and Luteyn, 1978; Jin et al., 2010; Wagstaff et al., 2010). Despite the ecological importance of leaf area, this variable was excluded from the study due to frequent visible damage on the leaves caused by herbivory or mechanical and environmental factors, which compromised the accuracy and comparability of the data. Therefore, the study prioritized reliable measurements by focusing on linear dimensions of the leaves. Also, from each individual, five randomly selected mature fruits were collected and used for morphological and physicochemical analyses, including longitudinal diameter (mm), width (mm), weight, Brix degrees, pH, and moisture content. Additionally, five extra fruits per plant were collected for manual seed extraction to avoid alterations in seed morphology due to the drying process. The total number of seeds per fruit was recorded. In total, 250 fruits per locality were evaluated for morphometric and physicochemical variables, and an additional 250 fruits were used for seed analysis. Moisture content was determined using the gravimetric method, following the AOAC (2000) protocol. Fruits were weighed upon arrival from the field (fresh weight), then dried in an oven at 100 °C for 24 hours until constant weight was achieved (dry weight). Moisture percentage was calculated using the following formula:


$$\text{Fruit humidity }\left(\text{H},\;\%\right)=\frac{Fresh\;weight-dry\;weight}{Fresh\;weight}\times 100$$

Climatic variables for each site were extracted from WorldClim raster layers (1 km² resolution) using the plant coordinates and the extract function from the ‘raster’ package in R (Fick and Hijmans, 2017). These environmental variables were later used to assess their relationship with the most influential morphological traits.

### 2.3 Data analysis

#### 2.3.1 Identification and validation of morphological groups of *Macleania rupestris*

To address whether *Macleania rupestris* populations can be grouped based on their most relevant morphological traits, we implemented a structured workflow consisting of data preprocessing, exploratory and confirmatory clustering, variable importance assessment, and functional characterization of trait divergence.

To ensure variable independence in subsequent analyses, a correlation analysis was conducted on the morphological trait dataset. Traits with a correlation coefficient of r ≥ 0.7 were removed using the findCorrelation function from the ‘caret’ package in R (Nukui and Onogi, 2023). The remaining data were then normalized using the Hellinger transformation with the decostand function from the ‘vegan’ package, minimizing the influence of differences in units and measurement scales (Oksanen et al., 2025). Next, an exploratory hierarchical clustering was conducted using Ward’s method and two distance metrics: Bray-Curtis and Euclidean. The clustering procedure was performed with the hclust function from the ‘cluster’ package (Maechler et al., 2025). The optimal distance metric was selected based on the highest cophenetic correlation, while the optimal number of clusters was determined using the elbow and Silhouette methods, implemented through the fviz_nbclust function from the ‘factoextra’ package (Kassambara and Mundt, 2020). Dendrograms representing the optimal clustering solution were generated using the dendextend and circlize functions. To statistically validate the resulting clusters, a Multivariate Analysis of Variance (MANOVA) was performed using the manova function, applying the Hotelling-Lawley test to assess statistical differences among groups. Once the most relevant traits were determined (see below), a second clustering was performed using the same method and metrics, to refine group classification. The resulting clusters were labeled and linked to each observation in a new matrix field.

To identify the most relevant traits contributing to the separation of the groups, a Random Forest model was fitted using the ‘randomForest’ package in R (Breiman et al., 2018). The model was built with the randomForest function, set to perform classification with 500 trees (ntree = 500). Variable importance was assessed using the importance function, which calculates the mean decrease in the Gini index. This index quantifies each trait’s contribution to reducing node impurity in the decision trees, thereby reflecting its relevance for group differentiation. For clearer interpretation, the Gini index values were transformed into percentages. Traits with an importance threshold ≥ 0.3 were selected as the most influential and retained for subsequent analyses to evaluate the functional structure of intraspecific trait variation.

A non-metric multidimensional scaling (NMDS) analysis was conducted to visualize the spatial distribution of the clusters and examine how the most relevant variables were associated with each group. To compare medians between clusters, the Wilcoxon test was applied to the most relevant traits (SNF, H, FWD, FL, P) after assessing normality with the Shapiro–Wilk test and evaluating residuals using histograms and Q–Q plots. Since the traits did not follow a normal distribution, the Wilcoxon test was chosen as a nonparametric alternative. To complement these comparisons and evaluate the structure of intraspecific trait variation (ITV), we fitted linear mixed-effects models (LMMs) for each trait using morphological group as a random effect. These models were specified with random intercepts and implemented using the lmer function from the ‘lme4’ package in R (Bates et al., 2015). The total variance was decomposed into between-group and within-group components, and the percentage of variance attributable to group structure was calculated as:


$$\%\text{Variancegroup}=\frac{\sigma 2\text{group}}{\sigma 2\text{group}+\sigma 2\text{residual}}\times 100$$

This approach allows quantifying the extent to which trait variation is structured among groups versus within them, providing a functional interpretation of ITV patterns (Albert et al., 2010; Siefert et al., 2015). For ecological interpretation, values above 70% were considered indicative of strong intergroup functional differentiation, values between 50% and 70% indicated moderate structuring, and values below 30–40% suggested high intra-group variability and low intergroup differentiation. These thresholds are not fixed standards, but rather serve as pragmatic guidelines to facilitate the interpretation of variance partitioning outcomes. Similar approaches have been used in previous studies analyzing intraspecific trait variation using hierarchical or mixed-effects models (e.g., Albert et al., 2010; Siefert et al., 2015).

Additionally, descriptive statistics (mean, standard deviation, minimum, maximum) and the coefficient of variation (CV) were calculated in general and for each trait within each group to assess the magnitude of ITV. To complement the NMDS, a Principal Component Analysis (PCA) was conducted using the five most relevant traits. Although NMDS was used for group visualization, PCA was implemented to generate synthetic trait axes summarizing the main dimensions of covariation. Since the data had already been normalized with the Hellinger transformation, the PCA was performed using the prcomp function with scaling disabled (scale. = FALSE). Trait contributions to the first two components (PC1 and PC2) were examined to interpret the functional basis of group differentiation. Additionally, the scores of PC1 were later used as a synthetic trait gradient in the modeling of environmental influences (see next section). Trait contributions to the first two components (PC1 and PC2) were examined to interpret the functional basis of group differentiation. All visualizations were generated using the ‘factoextra’ (Kassambara and Mundt, 2020) and ‘ggplot2’ (Wickham, 2016) packages.

#### 2.3.2 Relationship between environment variables and morphological groups

To establish relationships between the traits (normalized using the Hellinger method) and the climatic variables, generalized linear models with Negative Binomial, Gaussian, Poisson, and Gamma distributions were fitted using the glm function. The best model was selected based on the lowest ratio of deviance to residual degrees of freedom. Additionally, a correlation analysis was conducted to remove climatic variables with r ≥ 0.7. using the findCorrelation function from the caret package in R (Nukui and Onogi, 2023) to avoid collinearity in the models. In this analysis, we also incorporated dispersal and habitat disturbance, both represented by the spatial autocorrelation of plant locations. To achieve this, a Principal Coordinates of Neighbor Matrices (PCNM) analysis was performed using the geographic coordinates of the plant locations, applying the pcnm function from the ‘vegan’ package in R (Oksanen et al., 2025). The function returns a set of spatial eigenvectors derived from the principal coordinates of a truncated distance matrix, which capture spatial structures at different scales and can be used as explanatory variables in ecological models (Borcard and Legendre, 2002). These spatial eigenvectors effectively act as proxies for spatially structured environmental variation, including potential anthropogenic disturbances and dispersal limitations, which often generate non-random spatial patterns in ecological data (Monti and Legendre, 2009; Lorente et al., 2013; Parreira et al., 2023). For the model, only the eigenvectors that showed statistical significance (*P<* 0.05) during the generation process were selected.

To further evaluate the influence of environmental gradients on overall morphological variation, we extracted the scores of the first principal component (PC1) from the PCA and used them as a synthetic response variable in a separate model. This was done to capture the main dimension of intraspecific trait variability (IVT), as PC1 accounted for over 60% of the total trait variation and integrated the most ecologically relevant traits. A generalized linear model with Gaussian distribution was then fitted using PC1 as the dependent variable and the same set of uncorrelated climatic and spatial predictors. This approach is widely validated in trait-based ecology, as PCA axes summarize orthogonal dimensions of trait covariation and provide robust, interpretable gradients for modeling trait–environment relationships (Peres-Neto et al., 2003; Pierick et al., 2024).

#### 2.3.3 Development of a digital model for classifying new plants into morphological groups

A machine learning approach using the Random Forest algorithm was implemented due to its robustness in handling complex, non-linear relationships and its capacity to identify the most influential variables driving morphological variation. The analysis was performed in R, and the morphological traits were normalized using the Hellinger transformation prior to model fitting. The model was trained with the randomForest function from the randomForest package in R (Breiman et al., 2018), using morphological group assignment as the response variable and 500 trees (ntree = 500). The number of variables randomly selected at each split was set to pp, corresponding to the number of key predictor variables.

Only the most relevant traits contributing to the separation of the two morphological groups were considered, based on their importance as quantified by the Gini index (see Section 2.3.1). Model performance was assessed using 10-fold cross-validation implemented through the caret package in R (Nukui and Onogi, 2023). Classification accuracy was calculated for each fold, and the mean and standard deviation were used to evaluate consistency across iterations. The final model was saved as an.RData object to ensure reproducibility. Additionally, model accuracy was assessed using the full training dataset to evaluate its ability to recover known group structures. This internal validation is useful in exploratory analyses, especially when no independent dataset is available (Ullmann et al., 2022). To further assess potential overfitting, the model was also evaluated using an independent test dataset created by randomly partitioning 70% of the data for training and 30% for testing. Accuracy on the test set was compared to the cross-validated accuracy from the training phase, and both metrics were visualized to assess the model’s generalization ability. A large discrepancy would indicate overfitting, while similar performance supports the model’s robustness. Although internally validated, the model is designed to classify new plants from external datasets into the predefined morphological groups, enabling its future application to field data across the species’ distribution.

## 3 Results

### 3.1 Exploratory cluster analysis and the most important morphological traits of *Macleania rupestris*

**Figure 2:**
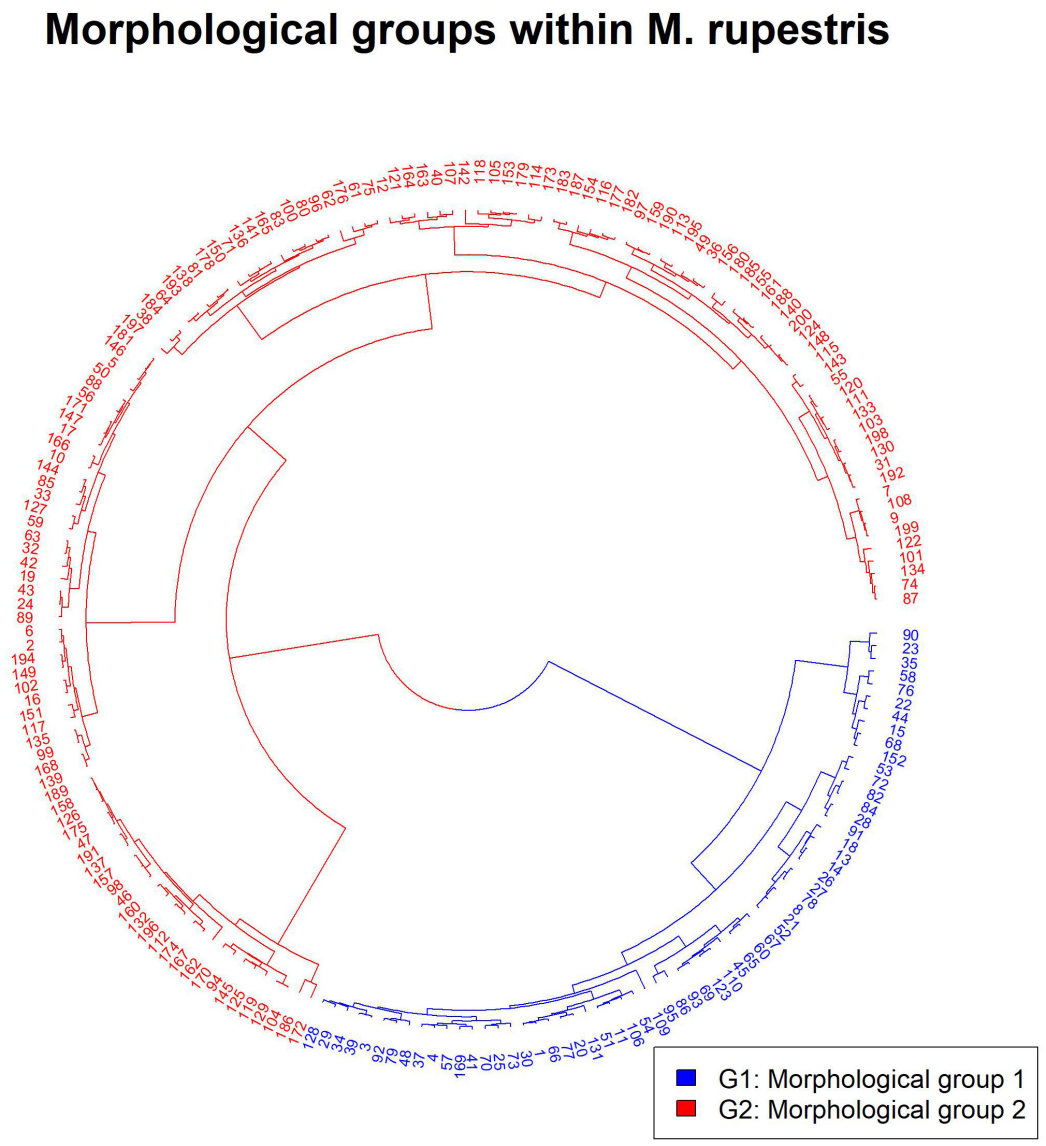
Cluster (Euclidean, Ward) showing the two morphological groups of plants for *M. rupestris*.

**Figure 3:**
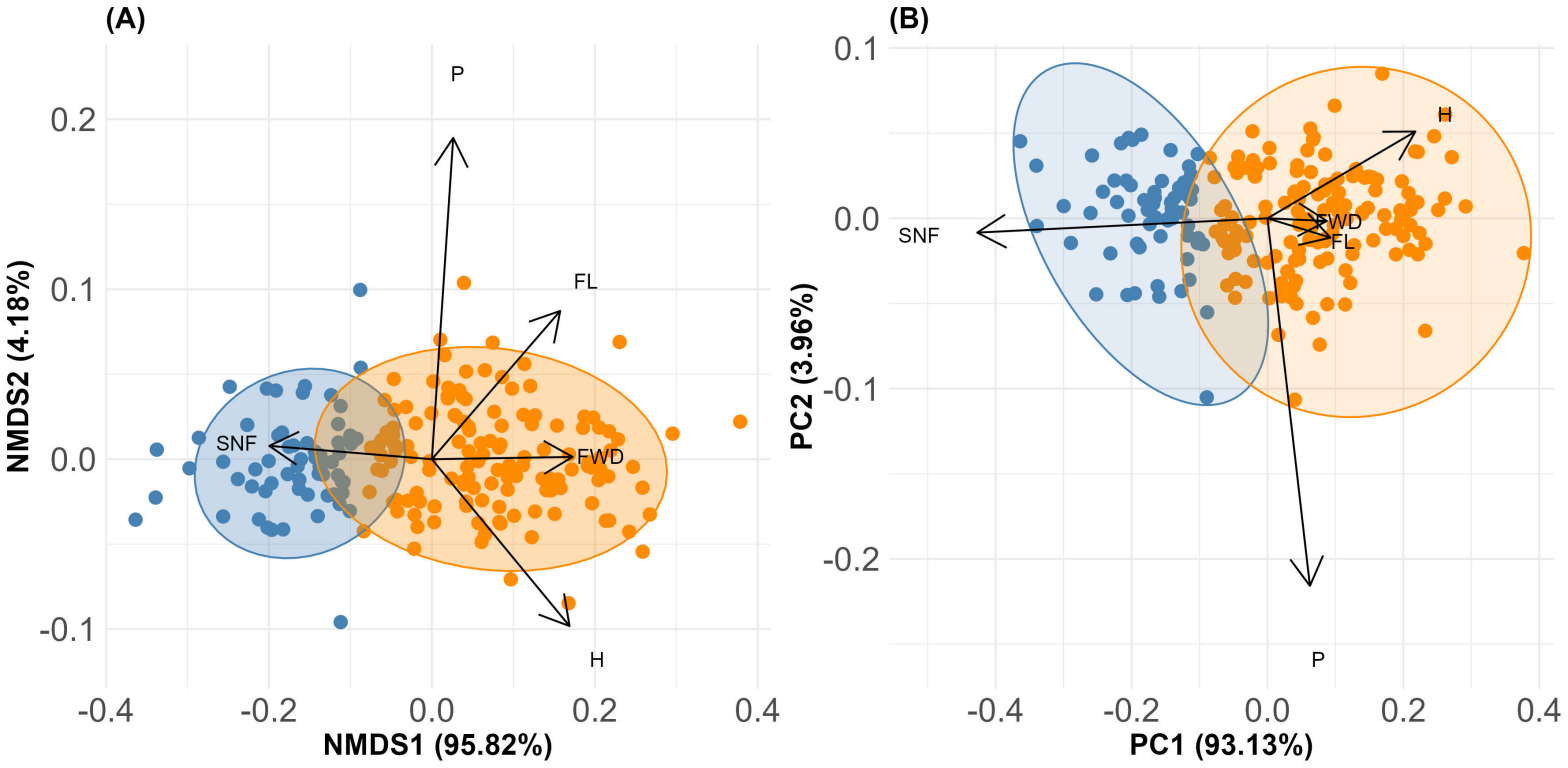
**(A)** NMDS ordination illustrating the spatial distribution of the two morphological groups of *M. rupestris*, based on the most relevant traits: number of seeds per fruit (SNF), fruit humidity (H), fruit width (FWD), fruit length (FL), and petiole length (P). **(B)** PCA summarizing the major morphological variation, with PC1 representing the axis of greatest variation and associated key traits.

None of the measured morphological traits were significantly correlated (r ≥ 0.7). The exploratory cluster analysis (**Supplementary Figure 2**), supported by the elbow test (**Supplementary Figure 3**) and the silhouette method (**Supplementary Figure 4**), identified two distinct groups within the *M. rupestris* populations: morphological group 1 (G1) and morphological group 2 (G2). These groups exhibited significant differences according to MANOVA (P< 0.0001). The most relevant traits contributing to group differentiation were: number of seeds per fruit (SNF), fruit humidity (H), fruit width (FWD), fruit length (FL), and petiole length (P), collectively accounting for over 75% of the variation (according to the Gini index transformed into percentage). These morphological traits supported the formation of the distinct cluster presented in **Figure 2**. The base matrix, comprising the most relevant traits and the groups corresponding to each observed plant, is presented in **Supplementary Table 2**. The NMDS analysis recorded a stress value of 0.3, indicating a valid separation of the two groups along the first axis that explained 95.82% of the variation (**Figure 3A**). Additionally, the results showed that SNF was strongly associated with Group 1, while FWD, FL, and H were associated with Group 2. In contrast, P exhibited an indifferent association between the groups.

The comparison of trait medians between the two morphological groups of *M. rupestris* revealed significant differences in several key traits (**Table 1**). G1 exhibited a significantly higher SNF than G2 (68.4 vs. 25.8; *P*< 0.0001). Conversely, H and FL were greater in G2 (83% and 13.1 mm, respectively) than in G1 (81.8% and 12.7 mm; *P* = 0.003 and *P* = 0.017, respectively). Although FWD was slightly higher in G2, the difference was not statistically significant (*P* = 0.182). P showed a borderline significant difference between groups (5.8 mm in G1 vs. 6.3 mm in G2; *P* = 0.046), suggesting a possible trend worth exploring in future studies.

**Table 1:** Medians and interquartile ranges (IQR) of the most important traits differentiating the morphological groups of *M. rupestris*, based on measurements from 200 plants.

Variable	Median G1	IQR G1	Median G2	IQR G2	*P*
SNF (number)	68.4 a	33.4	25.8 b	15.9	<0.0001
H (%)	81.8 a	4.3	83 b	3.2	0.003
FWD (mm)	13.5 a	1.8	14 a	2.1	0.182
FL (mm)	12.7 a	1.7	13.1 b	2.1	0.017
P (mm)	5.8 a	2.5	6.3 b	2.6	0.046

G1, Morphological group 1; G2, Morphological group 2; SNF, number of seeds per fruit; H, fruit humidity; FWD, fruit width; FL, fruit length; P, petiole length.

The most relevant traits exhibited varying degrees of intraspecific variation. Among these, the SNF showed the greatest variability, contrasting with the relative stability of H. FWD and fruit length FL presented moderate variation, while petiole length P showed intermediate variability. Differences between morphological groups were most evident in seed number and associated variability, whereas fruit humidity remained consistent across groups. Detailed descriptive statistics for these and other measured traits are available in **Supplementary Table 3**.

In addition to the comparison of medians, the structure of intraspecific trait variation (ITV) was evaluated by partitioning the total variance into between-group and within-group components for each trait. The proportion of variance attributable to group identity ranged from 28.5% for P to 85.6% for H (**Table 2**). The highest group-level variance components were observed for H (85.6%), SNF (82.7%), FL (70.5%) and FWD (64.1%), while P exhibited a lower proportion of between-group variance.

**Table 2:** Variance partitioning results for five functional traits of *M. rupestris*.

Variable	Group-level variance	Residual variance	% Variance attributable to group
SNF	0.0695	0.0145	82.7%
H	0.0262	0.0044	85.6%
FWD	0.0007	0.0004	64.1%
FL	0.0008	0.0003	70.5%
P	0.0002	0.0005	28.5%

Values represent the variance components estimated from linear mixed-effects models with morphological group as a random effect.

SNF, number of seeds per fruit; H, fruit humidity; FWD, fruit width; FL, fruit length; P, petiole length.

The percentage of variance attributable to group identity indicates the relative contribution of between-group variation to total trait variability.

### 3.2 Relationship between environmental variables and morphological groups

With respect to the uncorrelated variables, climatic seasonality and spatial autocorrelation significantly influenced the main morphological traits used for classification (**Table 3**). Temperature seasonality showed a positive effect on H and FL, but a negative effect on the number of SNF. Similarly, precipitation seasonality had a significant positive effect on H, FL, and P, but negatively affected SNF. Spatial autocorrelation, representing dispersal processes and potentially habitat disturbance, exerted a negative influence on H and FL, while positively affecting SNF and P. These findings highlight the role of climatic variability and spatial processes in determining variation in seed number, humidity, and fruit size, key traits linked to adaptation. FWD was not evaluated further, as it exhibited no significant differences between morphological groups (**Table 1**).

**Table 3:** GLM to relate the most important traits in the morphological groups of *M. rupestris* with climate and dispersal variables (spatial autocorrelation).

Predictor variables	H		SNF		FL		P		PC1	
	E	*P*	E	*P*	E	*P*	E	*P*	E	*P*
(Intercept)	0.11	0.246	1.82	<0.0001	-0.11	0.0074	-0.09	0.07	-1.52	<0.0001
Temperature Seasonality (standard deviation ×100)	0.003	0.0004	-0.006	<0.0001	0.002	<0.0001	0.0007	0.05	0.006	<0.0001
Precipitation Seasonality (Coefficient of Variation)	0.012	<0.0001	-0.04	<0.0001	0.011	<0.0001	0.009	<0.0001	0.042	<0.0001
Spatial autocorrelation (dispersal)	-0.17	0.006	0.28	0.0129	-0.033	0.2188	0.12	0.0002	0.305	0.0184

E, Coefficient estimator; *P*, p-value (95% probability).

In addition, the PCA axis PC1, which summarized the major variation in the morphological dataset, particularly in traits such as SNF, H, FWD, and fruit length FL (**Figure 3B**), was significantly explained by the same environmental predictors, with precipitation and temperature seasonality as well as spatial autocorrelation related to dispersal and habitat disturbance, all showing strong effects (**Table 3**). This reinforces the association between environmental gradients, particularly those shaped by climatic seasonality and habitat disturbance, and trait-based group differentiation.

### 3.3 Models to determine the correspondence of new plants to the morphological groups of *Macleania rupestris*

**Figure 4:**
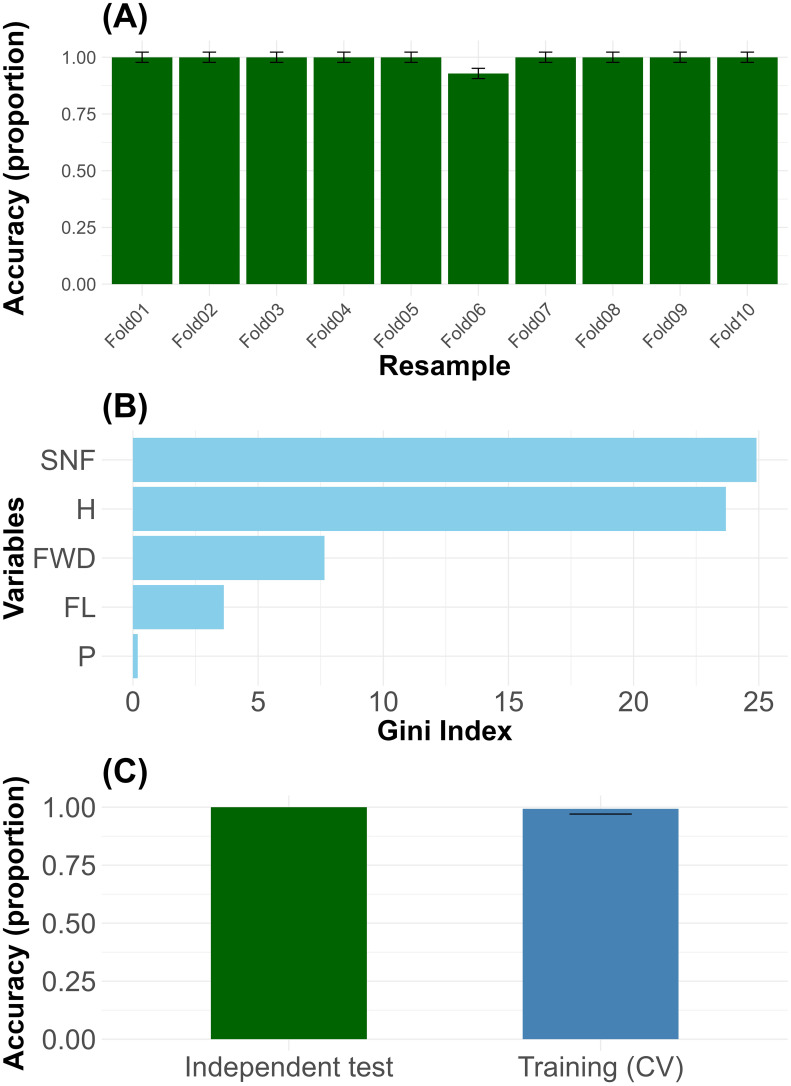
Mean classification accuracy and variability across 10-fold cross-validation folds **(A)**, variables importance ranked by mean decrease in Gini index across morphological traits **(B)**, and accuracy of the Random Forest model during cross-validation and independent testing, showing no evidence of overfitting **(C)**.

The Random Forest model exhibited excellent predictive performance, achieving an overall classification accuracy of 99.5%, corresponding to a misclassification rate of only 0.5%. The confusion matrix indicated that 99.49% of the original observations were correctly assigned to their respective groups. When applied to the original dataset, the model reached a perfect prediction accuracy of 100%. The 10-fold cross-validation procedure demonstrated consistent accuracy across all folds, with minimal variability in classification performance (**Figure 4A**). The importance of the variables evaluated through the average decrease in the Gini index identified the number of seeds per fruit (SNF; 24.9), followed by fruit humidity (H; 23.7), fruit width (FWD; 7.6), fruit length (FL; 3.6), and petiole length (P; 0.2). These findings are summarized in a composite figure (**Figure 4B**), which presents the contribution of each variable to the model. Collectively, these results confirm the robustness and reliability of the model for classifying individuals based on key morphological traits. The accuracy obtained during cross-validation (CV) training was similar to that achieved on the independent test set, indicating that the Random Forest model does not exhibit overfitting (**Figure 4C**). **Supplementary Code** (Compressed folder in zip format) provides the R code employed for model construction, classification of new plants, and evaluation. Inside the folder, read the text note.

## 4 Discussion

### 4.1 Morphological variation in *Macleania rupestris*

In this study, the intraspecific variation of *Macleania rupestris* was evident, as populations were successfully grouped based on morphological traits. The exploratory cluster analysis, supported by the elbow and silhouette methods, identified two distinct morphological groups (G1, G2) within the *M. rupestris* populations. These groups exhibited significant differentiation confirmed by MANOVA (P< 0.0001), highlighting clear morphological structuring. Notably, none of the measured morphological traits were strongly correlated (r ≥ 0.7), suggesting that the traits assessed contribute independently to the observed variation and group differentiation.

Group 1 exhibiting a higher number of seeds per fruit, while Group 2 consisted of taller plants with larger fruits. This pattern aligns with previous studies demonstrating that seed number and fruit size are key traits for distinguishing groups within a species (Ashok, 2000; Monforte et al., 2013). The observed differentiation underscores the importance of analyzing multiple morphological traits to assess intraspecific variation, a widely used approach in plant taxonomy and classification (Cope et al., 2012; Tsakalos et al., 2019). Similar trends have been reported in *Carya laciniosa*, where seed and fruit traits influence dispersal efficiency, germination rates, and seedling establishment, ultimately shaping population structure and local adaptation (Primack, 1987; Bonner, 2008). These findings reinforce that the differences in growth and reproductive traits highlighted by the NMDS analysis reflect consistent morphological divergence among populations, likely shaped by ecological or genetic factors driving local adaptation.

The most relevant morphological traits, such as number of seeds per fruit (SNF), fruit humidity (H), fruit width (FWD), fruit length (FL), and petiole length (P), likely not only structure morphological variability but are also closely linked to key ecological processes such as reproduction, dispersal, and adaptation to the microenvironment. SNF is a direct indicator of reproductive success and the potential for colonization of new areas, as it influences dispersal efficiency and seedling establishment capacity (Rodriguez-García et al., 2019; Leal and Koski, 2023). H, in turn, affects seed viability and dispersal timing, reflecting adaptations to variable water regimes and resource availability in the environment (Baskin and Baskin, 2000). FWD and FL influence interactions with frugivores and dispersal mechanisms, affecting the distance and pattern of seed dispersal (Mazer and Wheelwright, 1993; Sobral et al., 2010). P may be related to fruit exposure and light capture efficiency, which also influence microenvironmental adaptation.

Variance partitioning of intraspecific trait variation (ITV) in *Macleania rupestris* demonstrated marked differences in the extent to which traits vary between versus within morphological groups. H showed a predominant between-group variance (85.6%), indicating that this trait is strongly conserved within groups but differs markedly among groups, reflecting possible adaptive divergence to local environmental conditions (Violle et al., 2012). In contrast, P exhibited a low proportion of between-group variance (28.5%), implying most variability arises within groups, consistent with traits that display phenotypic plasticity responding probably to microhabitat heterogeneity (Galloway, 1995; Sultan, 2000; Baythavong and Stanton, 2010). Key reproductive and dispersal traits such as SNF, FL, and FWD also showed substantial between-group differentiation (64.1%–82.7%), underscoring their role in local adaptation and divergent selection pressures shaping population structure (Vellend et al., 2006; Siefert et al., 2015; Nicolaus and Edelaar, 2018). Together, these findings illustrate a balance in *M. rupestris* between strong adaptive trait divergence among populations and plasticity within populations, mechanisms that likely enhance persistence and fitness in variable montane environments. Furthermore, the combined use of cluster analysis, NMDS, and variance partitioning provides a comprehensive framework to understand the morphological and ecological underpinnings of intraspecific variation in *M. rupestris*, emphasizing the role of reproductive and dispersal-related traits in shaping population differentiation and local adaptation.

### 4.2 Environmental variables and morphological groups of Macleania rupestris

Climatic seasonality strongly influences coordinated morphological trait variation in *Macleania rupestris*, with temperature and precipitation regimes modulating reproductive and structural traits in concert. For instance, temperature seasonality positively affected fruit humidity (H) and fruit length (FL), while negatively influencing seed number per fruit (SNF), reflecting possible reproductive trade-offs under fluctuating thermal conditions (Hedhly et al., 2009; Tushabe et al., 2023). Similarly, precipitation seasonality showed positive effects on H, FL, and petiole length (P), but reduced SNF indicating a potential association between rainfall variability and patterns of resource allocation and fruit development. These patterns are consistent with evidence that high climatic seasonality induces reproductive stress, disrupts flowering phenology, and reduces seed set (Fenner and Thompson, 2005; Richardson et al., 2013; Tunes et al., 2017). In contrast, the relatively stable climatic conditions of humid montane forests promote more robust fruit traits and higher seed production by minimizing stress and optimizing pollinator and disperser activity (Chazdon and Arroyo, 2013; Zhang et al., 2022). Consequently, climate-driven variation in reproductive and vegetative traits reflects adaptive responses to environmental stability, matching patterns found in other tropical plant species (Hedhly et al., 2009; Wright et al., 2010; March-Salas et al., 2021).

Importantly, Principal Component Analysis (PCA) identified the first principal component (PC1) as the major axis summarizing over 60% of morphological variation among groups, with strong loadings from SNF (–0.85), H (0.43), FL (0.18), and fruit width (FWD; 0.17). By using PC1 scores in subsequent generalized linear models, we robustly assessed how climatic and spatial factors jointly shape intraspecific trait variability (ITV), moving beyond trait-by-trait analysis toward an integrated functional perspective (Peres-Neto et al., 2003; Pierick et al., 2024). This revealed that ITV in *M. rupestris* is structured and functionally coordinated, reflecting adaptive morphological strategies to cope with climatic stress and spatial dispersal constraints. These patterns are consistent with those described in other plant systems (Wright et al., 2010; Violle et al., 2012), suggesting that the coordination of trait variation in response to environmental heterogeneity may represent a general ecological mechanism, now also evidenced in a tropical montane shrub. Spatial autocorrelation due to limited dispersal showed contrasting influences on traits, positively affecting SNF and P but negatively impacting H and FL, indicating that localized recruitment influences both reproductive and structural investments at the population level (Matthysen, 2012; Mathur, 2015). Together, these findings underscore the complex interaction between climate and spatial processes driving morphological differentiation and functional trait variability within *M. rupestris* populations.

Although *M. rupestris* is frequently observed in disturbed environments, such as forest edges and regenerating areas, our study did not explicitly quantify disturbance metrics (e.g., canopy openness, land-use intensity). However, spatial eigenvectors derived from PCNM analysis were included in our models to account for broad-scale spatial patterns. While originally implemented to capture dispersal-related structure, PCNM variables are also known to reflect spatially structured environmental gradients, including those generated by anthropogenic disturbance (Borcard and Legendre, 2002; Dray et al., 2006). As such, they provide a reasonable spatial proxy for unmeasured heterogeneity when field-based disturbance data are lacking. In our case, the significant effects of these spatial predictors on key traits suggest that they may also be capturing latent signals of habitat disturbance across the landscape. While not a substitute for direct measurements, their inclusion offers an indirect yet informative way to account for disturbance-related variability in ITV. Future research would benefit from integrating explicit disturbance metrics to refine interpretations of trait-environment relationships in the context of land-use dynamics.

### 4.3 Predictive model

The high classification accuracy of the Random Forest model (99.5%) highlights the presence of consistent and biologically meaningful morphological differences among groups of *Macleania rupestris*, suggesting ecological differentiation likely associated with environmental heterogeneity. The model’s ability to handle complex ecological data with a low error rate, as previously demonstrated in vegetation studies (Cutler et al., 2007; Belgiu and Drăguţ, 2016), reinforces the robustness of our classification. Seed number per fruit (SNF) and fruit humidity (H) emerged as the most influential traits, corroborating their relevance in morphological group differentiation (Liaw and Wiener, 2002). Their high mean decrease in the Gini index supports their role in defining key axes of divergence. Although fruit length (FL), fruit width (FWD), and petiole length (P) were less influential, they still contributed to capturing relevant variation. Overall, the pattern of trait importance reflects biologically meaningful morphological variation, potentially shaped by ecological conditions and local environmental pressures.

Although the model’s performance was evaluated through internal cross-validation, it was specifically designed for external datasets. The accompanying R code and classification model are fully operational and can be used to classify *M. rupestris* individuals from unsampled populations based on the same morphological traits. This allows researchers, conservation practitioners, and restoration planners to validate the model with their field data, extending its applicability across the species’ range.

### 4.4 Intraspecific vs. interspecific trait variation in functional ecology

Our findings emphasize the ecological relevance of intraspecific trait variability (ITV) in *Macleania rupestris*, as trait divergence among morphological groups was strongly structured by environmental gradients. While this study focused on ITV, it is important to contextualize these results within the broader framework of functional ecology, where both intraspecific and interspecific variability (ETV) contribute to ecosystem functioning. Recent research has emphasized that ITV can equal or even surpass ETV in magnitude, especially in heterogeneous environments where plastic and adaptive responses to local conditions are critical (Siefert et al., 2015; Banitz, 2019; Puglielli et al., 2024). In this context, ITV is not merely noise within species but represents a fundamental component of community-level trait distributions, influencing processes such as niche differentiation, community assembly, and ecosystem resilience (Westerband et al., 2021; Palacio et al., 2025). The strong ITV patterns observed in key functional traits of *M. rupestris* (e.g., SNF, H, FL) highlight the importance of intra-population variability in structuring morphological responses to environmental gradients in montane systems characterized by high environmental turnover. A better understanding of the balance between ITV and ETV in such systems will help refine predictive models of trait-environment relationships and inform biodiversity conservation under climate change scenarios.

## 5 Conclusions and implications for management

The results of this study reveal clear intraspecific morphological variation in *Macleania rupestris*, with two morphologically different groups (G1 and G2) associated with climatic gradients and spatial patterns. Traits such as seed number per fruit (SNF), and fruit length (FL) explained over 75% of the variation between groups and were significantly associated with environmental variables, including temperature and precipitation seasonality. The high accuracy of the Random Forest classification model (99.5%) underscores its potential as a robust tool for morphological monitoring, the identification of locally adaptive units, and the development of evidence-based conservation strategies. Number of seeds per fruit and fruit humidity were the most discriminating variables in this model, while fruit length, fruit width, and petiole length were less important. These findings highlight the relevance of integrating key reproductive and vegetative traits in intraspecific analyses, supporting their application in future ecological differentiation studies and conservation planning across heterogeneous landscapes.

Based on these findings, complementary strategies are proposed to support the conservation of *M. rupestris.* One strategy aims to ensure the *in-situ* conservation of both identified morphological units, safeguarding their morphological diversity and adaptive potential. A second strategy focuses on the operational use of the classification model in ecological restoration initiatives. This includes the identification of locally adapted individuals, the collection and propagation of traceable plant material, targeted planting according to site-specific environmental conditions, and subsequent monitoring to inform adaptive management. This integrative approach bridges applied science and restoration practice, strengthening the resilience of montane ecosystems in the face of environmental change and habitat fragmentation.

Future research should investigate whether the morphological groups identified here reflect underlying genetic differences. Although this study does not address molecular variation directly, it provides a morphological framework for future genetic or phylogeographic analyses assessing evolutionary uniqueness and adaptive relevance.

## Data Availability

The original contributions presented in the study are included in the article/**Supplementary Material**. Further inquiries can be directed to the corresponding authors.
