# Supplementary material for: Intraspecific morphological variation and environmental drivers in Macleania rupestris: a model-based population classification
Source: Front Plant Sci. 2025 Aug 12;16:1645659. doi: 10.3389/fpls.2025.1645659 (PMC12378330; doi:10.3389/fpls.2025.1645659)
Supplement: Supplementary file 1 [file Table1.docx]

**Intraspecific Morphological Variation and Environmental Drivers in *Macleania rupestris*: A Model-Based Population Classification**

# Supplementary Data

R code and any files attached to it are stored in a compiled (.zip) folder called Supplementary Code.

# Supplementary Figures and Tables

## Supplementary Figures


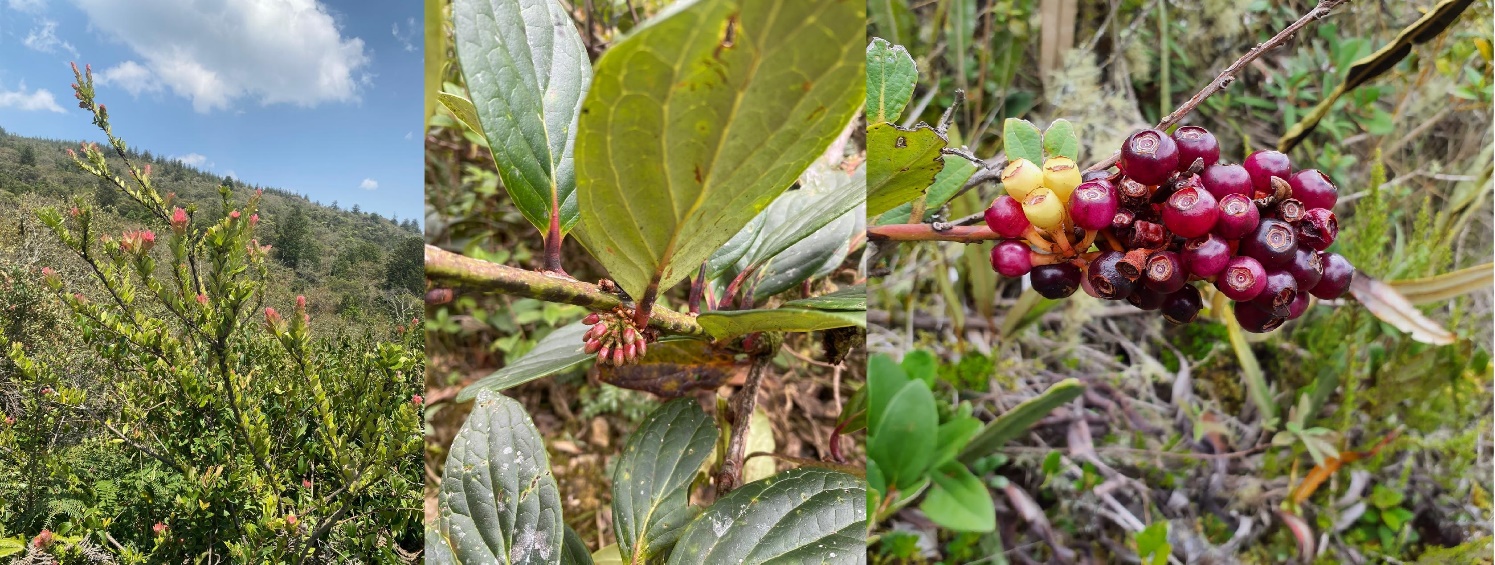


**Supplementary Figure 1.** *Macleania rupestris* growing in its natural montane forest habitat, showing its shrubby growth form, leaf arrangement, and characteristic fruit morphology.


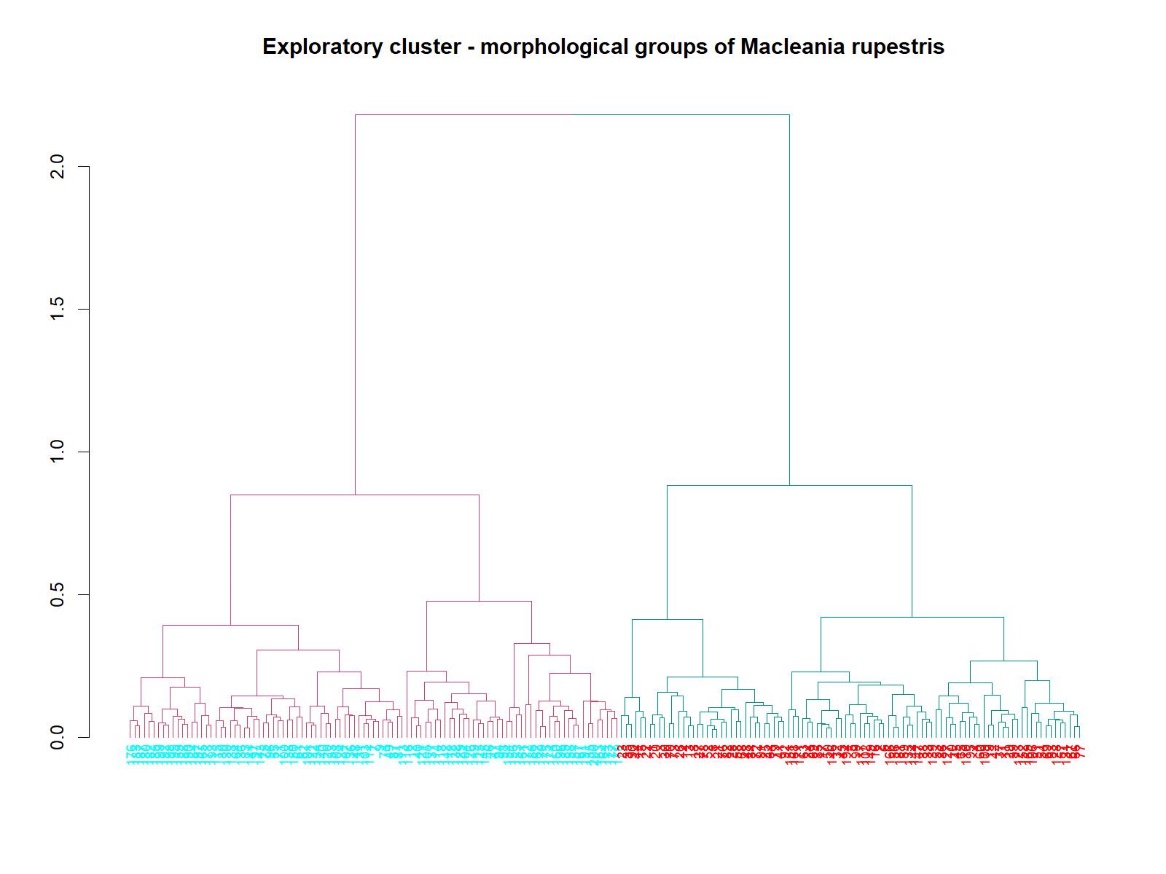


**Supplementary Figure 2.** Exploratory cluster analysis (Euclidean distance, Ward’s method) performed using all morphological traits measured in 200 *Macleania rupestris* individuals from four populations in southern Ecuador, aimed at identifying preliminary morphological groupings prior to trait selection.


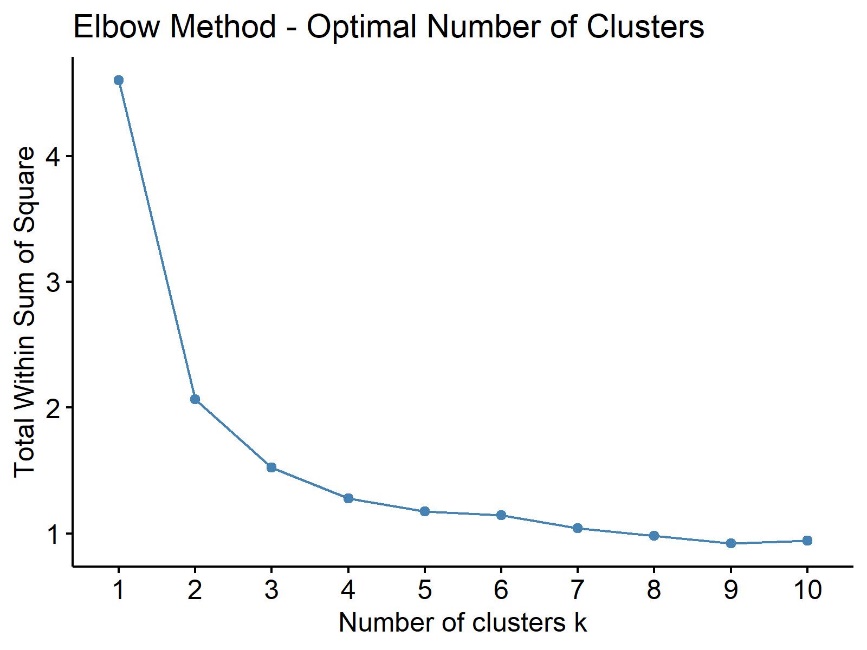


**Supplementary Figure 3.** Elbow method applied to determine the optimal number of clusters in the exploratory hierarchical analysis of *Macleania rupestris* individuals based on complete morphological trait data. The plot suggests the presence of two well-supported morphological groups.


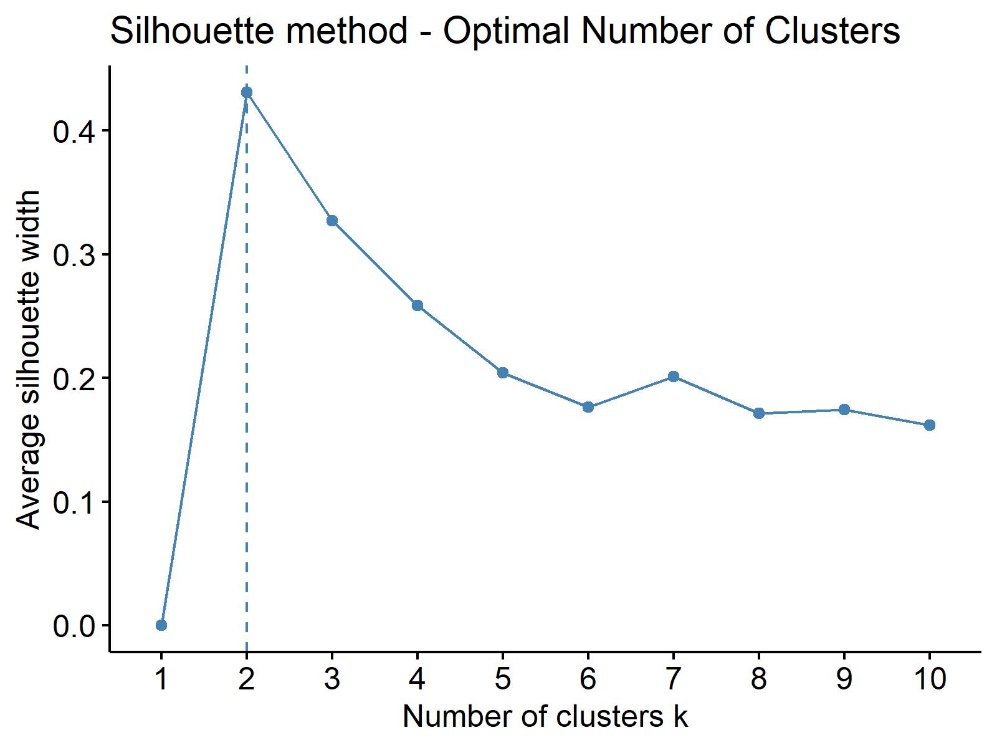


**Supplementary Figure 4.** Silhouette analysis used to validate the optimal number of clusters derived from exploratory hierarchical clustering of *Macleania rupestris*. The silhouette width supports a two-cluster solution, indicating good separation and cohesion of morphological groups.

**Supplementary Table 1.** Climatic variables of the study localities: Annual mean temperature (ºC), annual precipitation (mm), temperature seasonality (standard deviation ×100), and precipitation seasonality (coefficient of variation)

| **Localities** | **Annual Mean Temperature (ºC)** | **Annual Precipitation (mm)** | **Temperature Seasonality (standard deviation ×100)** | **Precipitation Seasonality (Coefficient of Variation)** |
| --- | --- | --- | --- | --- |
| Guel | 13.1 | 799.0 | 64.9 | 23.5 |
| Luis Cordero | 14.6 | 805.0 | 53.8 | 26.6 |
| Nabon | 10.0 | 1004.0 | 68.0 | 27.7 |
| San Vicente | 13.9 | 778.0 | 54.3 | 28.3 |

**Supplementary Table 2.** Data on 15 morphological traits of *M. rupestris* sampled from 200 individuals and used in exploratory analyses. The table includes the most important traits that contributed to the separation of morphological groups and were used in the Random Forest predictive model.

| **Site** | **Plant** | **P** | **LW** | **LeL** | **RL** | **FW** | **DFW** | **FL** | **FWD** | **SNF** | **PH** | **LL** | **FNB** | **FRB** | **H** | **BR** | **Group** |
| --- | --- | --- | --- | --- | --- | --- | --- | --- | --- | --- | --- | --- | --- | --- | --- | --- | --- |
| Guel | 1 | 5.8 | 3.0 | 6.4 | 1.2 | 2.3 | 0.3 | 14.6 | 15.5 | 80.2 | 0.8 | 1.3 | 16.0 | 12.5 | 85.2 | 10.6 | 1 |
| Guel | 2 | 4.5 | 3.1 | 5.6 | 0.9 | 1.3 | 0.2 | 12.2 | 12.9 | 46.4 | 1.3 | 2.3 | 14.0 | 8.5 | 83.1 | 12.9 | 1 |
| Guel | 3 | 5.8 | 2.8 | 6.6 | 0.9 | 3.3 | 0.2 | 12.0 | 12.4 | 77.6 | 1.6 | 1.8 | 15.0 | 7.8 | 93.1 | 11.4 | 1 |
| Guel | 4 | 6.3 | 3.4 | 7.2 | 2.9 | 1.2 | 0.2 | 11.4 | 13.4 | 74.4 | 2.1 | 1.6 | 13.5 | 11.3 | 81.9 | 11.0 | 1 |
| Guel | 5 | 7.0 | 3.6 | 7.2 | 0.9 | 1.4 | 0.2 | 12.6 | 13.3 | 52.2 | 2.2 | 1.7 | 16.0 | 6.3 | 85.4 | 10.9 | 1 |
| Guel | 6 | 4.5 | 2.5 | 6.1 | 1.1 | 1.0 | 0.1 | 11.9 | 12.0 | 45.6 | 2.2 | 1.3 | 12.5 | 8.8 | 86.9 | 11.7 | 1 |
| Guel | 7 | 4.3 | 2.7 | 4.9 | 0.7 | 1.3 | 0.2 | 11.9 | 13.1 | 31.4 | 2.2 | 1.9 | 13.5 | 11.3 | 85.9 | 13.0 | 2 |
| Guel | 8 | 5.0 | 3.0 | 8.4 | 1.5 | 1.4 | 0.2 | 12.4 | 13.4 | 97.0 | 2.2 | 2.1 | 16.0 | 11.3 | 84.0 | 14.8 | 1 |
| Guel | 9 | 3.8 | 2.4 | 6.4 | 1.1 | 1.5 | 0.3 | 12.1 | 13.8 | 29.4 | 1.4 | 2.0 | 16.3 | 13.8 | 83.3 | 13.8 | 2 |
| Guel | 10 | 5.8 | 3.1 | 7.5 | 1.2 | 2.1 | 0.4 | 13.1 | 15.4 | 55.6 | 2.3 | 1.9 | 14.8 | 12.5 | 80.2 | 10.4 | 1 |
| Guel | 11 | 4.8 | 2.8 | 7.6 | 0.8 | 1.4 | 0.2 | 12.9 | 13.3 | 68.0 | 2.4 | 2.6 | 17.0 | 13.3 | 85.2 | 10.8 | 1 |
| Guel | 12 | 7.0 | 3.6 | 8.5 | 0.8 | 1.3 | 0.2 | 11.9 | 12.3 | 17.0 | 1.1 | 2.0 | 17.0 | 11.8 | 85.2 | 12.2 | 2 |
| Guel | 13 | 3.5 | 3.6 | 7.1 | 0.9 | 1.3 | 0.2 | 12.4 | 13.0 | 101.4 | 1.4 | 1.9 | 13.8 | 10.3 | 84.8 | 10.2 | 1 |
| Guel | 14 | 3.0 | 3.3 | 5.5 | 0.6 | 1.5 | 0.3 | 12.7 | 13.4 | 90.6 | 1.6 | 1.4 | 14.3 | 10.0 | 77.2 | 15.7 | 1 |
| Guel | 15 | 3.5 | 2.3 | 5.1 | 1.0 | 2.4 | 0.5 | 13.5 | 16.1 | 119.0 | 1.6 | 1.2 | 14.5 | 10.0 | 78.7 | 14.2 | 1 |
| Guel | 16 | 4.3 | 3.4 | 7.0 | 0.7 | 1.3 | 0.2 | 12.7 | 12.5 | 39.0 | 1.3 | 1.8 | 10.3 | 8.0 | 80.7 | 9.6 | 1 |
| Guel | 17 | 6.8 | 3.2 | 7.0 | 1.1 | 1.4 | 0.3 | 11.6 | 13.0 | 53.2 | 1.3 | 1.0 | 16.0 | 11.8 | 78.8 | 14.9 | 1 |
| Guel | 18 | 3.3 | 2.4 | 5.4 | 0.6 | 1.3 | 0.2 | 11.3 | 13.4 | 87.8 | 1.4 | 1.4 | 13.5 | 9.0 | 83.4 | 9.4 | 1 |
| Guel | 19 | 4.3 | 3.3 | 5.7 | 1.4 | 1.2 | 0.2 | 9.0 | 12.8 | 57.0 | 1.8 | 1.4 | 15.0 | 11.0 | 83.8 | 10.6 | 1 |
| Guel | 20 | 5.0 | 2.1 | 6.0 | 1.0 | 1.9 | 0.3 | 13.0 | 14.5 | 66.8 | 0.9 | 1.8 | 14.8 | 8.5 | 81.2 | 10.2 | 1 |
| Guel | 21 | 4.5 | 3.4 | 9.0 | 1.7 | 1.9 | 0.4 | 12.8 | 14.9 | 96.4 | 2.4 | 2.3 | 16.0 | 11.0 | 79.0 | 15.3 | 1 |
| Guel | 22 | 5.3 | 3.8 | 7.7 | 0.8 | 2.1 | 0.5 | 13.2 | 15.3 | 138.6 | 2.1 | 3.0 | 15.0 | 10.5 | 75.4 | 15.6 | 1 |
| Guel | 23 | 3.0 | 2.6 | 5.1 | 0.8 | 1.8 | 0.3 | 12.7 | 14.7 | 188.8 | 1.0 | 1.7 | 13.5 | 9.8 | 82.8 | 14.5 | 1 |
| Guel | 24 | 4.5 | 3.4 | 6.1 | 1.8 | 1.5 | 0.3 | 12.1 | 13.7 | 50.8 | 4.1 | 1.5 | 15.5 | 13.5 | 83.6 | 11.9 | 1 |
| Guel | 25 | 5.5 | 3.0 | 5.4 | 0.8 | 1.7 | 0.3 | 12.5 | 14.2 | 89.2 | 2.9 | 1.0 | 12.0 | 9.3 | 82.4 | 8.9 | 1 |
| Guel | 26 | 3.3 | 4.8 | 7.8 | 0.6 | 1.8 | 0.3 | 14.6 | 14.4 | 95.6 | 0.9 | 2.5 | 10.0 | 6.0 | 82.6 | 12.5 | 1 |
| Guel | 27 | 4.5 | 2.9 | 5.9 | 1.2 | 1.2 | 0.3 | 11.4 | 12.4 | 99.6 | 2.3 | 1.3 | 16.8 | 13.0 | 78.7 | 12.7 | 1 |
| Guel | 28 | 10.3 | 4.0 | 7.3 | 1.0 | 2.1 | 0.4 | 14.4 | 15.0 | 104.8 | 1.5 | 1.3 | 16.0 | 11.3 | 82.1 | 9.5 | 1 |
| Guel | 29 | 4.8 | 2.7 | 6.8 | 0.7 | 0.9 | 0.2 | 10.9 | 11.4 | 64.4 | 2.3 | 1.5 | 16.0 | 12.8 | 72.2 | 9.8 | 1 |
| Guel | 30 | 5.8 | 3.3 | 6.8 | 0.6 | 2.3 | 0.5 | 14.4 | 15.5 | 74.6 | 2.4 | 1.8 | 11.5 | 7.8 | 80.0 | 9.3 | 1 |
| Guel | 31 | 4.8 | 2.6 | 4.8 | 1.9 | 1.9 | 0.2 | 14.1 | 14.4 | 35.6 | 1.6 | 1.5 | 16.5 | 17.5 | 87.9 | 12.2 | 2 |
| Guel | 32 | 5.8 | 1.0 | 6.4 | 0.9 | 1.2 | 0.2 | 11.4 | 12.8 | 53.0 | 1.5 | 2.5 | 18.0 | 12.8 | 81.7 | 14.8 | 1 |
| Guel | 33 | 8.0 | 3.6 | 8.7 | 1.0 | 1.4 | 0.3 | 11.4 | 13.6 | 45.2 | 2.6 | 1.3 | 15.0 | 9.3 | 81.8 | 14.7 | 1 |
| Guel | 34 | 4.8 | 2.4 | 4.8 | 0.9 | 0.9 | 0.2 | 10.7 | 11.2 | 75.4 | 2.9 | 1.1 | 13.0 | 7.8 | 81.3 | 8.7 | 1 |
| Guel | 35 | 4.0 | 3.2 | 5.9 | 0.9 | 1.7 | 0.3 | 14.7 | 13.4 | 175.0 | 3.2 | 1.5 | 16.5 | 10.5 | 84.0 | 11.9 | 1 |
| Guel | 36 | 6.8 | 4.3 | 8.8 | 0.9 | 1.1 | 0.2 | 11.2 | 12.5 | 25.8 | 2.3 | 2.7 | 11.0 | 9.0 | 84.3 | 12.4 | 2 |
| Guel | 37 | 5.8 | 3.3 | 7.3 | 0.9 | 1.9 | 0.4 | 11.1 | 14.9 | 73.8 | 2.5 | 2.8 | 15.5 | 15.8 | 80.3 | 12.4 | 1 |
| Guel | 38 | 9.5 | 3.2 | 7.4 | 2.2 | 2.4 | 0.5 | 14.8 | 16.2 | 42.8 | 1.4 | 1.9 | 17.0 | 10.5 | 79.9 | 14.0 | 2 |
| Guel | 39 | 5.0 | 2.3 | 6.0 | 1.2 | 1.0 | 0.2 | 11.2 | 11.8 | 68.4 | 1.4 | 1.5 | 18.3 | 16.0 | 84.2 | 13.3 | 1 |
| Guel | 40 | 4.5 | 3.6 | 7.3 | 1.2 | 1.0 | 0.2 | 10.8 | 11.4 | 19.8 | 2.0 | 2.1 | 17.0 | 10.0 | 78.3 | 13.5 | 2 |
| Guel | 41 | 6.5 | 2.6 | 6.2 | 1.1 | 2.0 | 0.3 | 13.2 | 14.9 | 88.0 | 2.0 | 2.3 | 18.0 | 11.0 | 86.1 | 9.9 | 1 |
| Guel | 42 | 4.8 | 2.4 | 6.4 | 0.9 | 1.3 | 0.2 | 12.2 | 12.6 | 57.0 | 1.9 | 1.7 | 14.5 | 10.3 | 82.9 | 11.1 | 1 |
| Guel | 43 | 4.3 | 2.0 | 9.5 | 0.8 | 1.5 | 0.2 | 12.3 | 13.8 | 50.4 | 1.7 | 1.2 | 20.0 | 13.3 | 85.4 | 14.0 | 1 |
| Guel | 44 | 7.8 | 2.6 | 6.8 | 1.4 | 2.4 | 0.4 | 14.4 | 15.6 | 147.8 | 1.0 | 1.4 | 21.3 | 14.0 | 83.8 | 10.0 | 1 |
| Guel | 45 | 7.5 | 3.2 | 7.0 | 1.2 | 2.4 | 0.4 | 13.3 | 16.4 | 68.4 | 1.7 | 1.5 | 15.0 | 12.8 | 84.3 | 12.4 | 1 |
| Guel | 46 | 7.3 | 2.4 | 5.8 | 0.8 | 1.8 | 0.3 | 13.7 | 14.0 | 11.6 | 1.8 | 2.6 | 17.3 | 11.3 | 81.3 | 14.3 | 2 |
| Guel | 47 | 6.3 | 2.4 | 6.4 | 1.2 | 1.6 | 0.2 | 12.5 | 13.7 | 13.6 | 1.4 | 1.2 | 15.8 | 10.8 | 86.7 | 14.8 | 2 |
| Guel | 48 | 6.3 | 3.2 | 5.6 | 1.3 | 0.8 | 0.1 | 10.1 | 10.8 | 86.2 | 2.0 | 5.8 | 21.0 | 9.3 | 86.3 | 9.6 | 1 |
| Guel | 49 | 8.3 | 3.0 | 6.4 | 1.3 | 1.0 | 0.2 | 10.7 | 11.6 | 28.6 | 2.5 | 1.7 | 18.3 | 12.0 | 84.6 | 10.8 | 2 |
| Guel | 50 | 7.8 | 2.6 | 6.2 | 1.2 | 1.3 | 0.2 | 10.8 | 13.8 | 51.8 | 2.0 | 1.5 | 17.8 | 14.0 | 83.9 | 15.1 | 1 |
| Luis Cordero | 51 | 4.5 | 3.5 | 7.6 | 1.6 | 1.2 | 0.2 | 12.5 | 12.2 | 68.2 | 2.0 | 2.4 | 14.3 | 6.8 | 85.9 | 14.4 | 1 |
| Luis Cordero | 52 | 6.3 | 3.9 | 9.0 | 1.5 | 2.1 | 0.3 | 13.9 | 15.0 | 102.0 | 1.9 | 3.1 | 14.8 | 9.8 | 83.9 | 16.0 | 1 |
| Luis Cordero | 53 | 10.0 | 4.5 | 7.8 | 1.5 | 1.8 | 0.3 | 14.6 | 14.0 | 85.2 | 5.2 | 3.1 | 14.3 | 6.0 | 81.3 | 12.5 | 1 |
| Luis Cordero | 54 | 4.0 | 3.5 | 7.3 | 1.9 | 1.5 | 0.2 | 12.7 | 13.5 | 64.2 | 2.8 | 2.3 | 13.5 | 8.5 | 84.1 | 19.6 | 1 |
| Luis Cordero | 55 | 5.5 | 2.9 | 7.5 | 0.9 | 1.5 | 0.2 | 12.5 | 13.5 | 34.8 | 2.6 | 1.8 | 9.3 | 6.0 | 85.0 | 14.0 | 2 |
| Luis Cordero | 56 | 7.3 | 3.8 | 8.0 | 2.4 | 2.2 | 0.4 | 14.2 | 15.2 | 54.0 | 2.9 | 1.9 | 11.3 | 8.5 | 84.3 | 15.8 | 1 |
| Luis Cordero | 57 | 6.3 | 3.8 | 8.1 | 1.1 | 2.1 | 0.4 | 12.8 | 15.0 | 77.8 | 2.9 | 1.5 | 16.5 | 10.3 | 81.2 | 17.8 | 1 |
| Luis Cordero | 58 | 10.5 | 3.5 | 7.9 | 1.4 | 1.7 | 0.3 | 12.4 | 14.0 | 123.8 | 4.4 | 2.3 | 15.5 | 10.0 | 82.4 | 15.1 | 1 |
| Luis Cordero | 59 | 9.3 | 3.7 | 5.2 | 0.8 | 2.4 | 0.4 | 14.3 | 15.5 | 56.8 | 2.5 | 2.1 | 11.0 | 9.5 | 82.3 | 17.0 | 1 |
| Luis Cordero | 60 | 8.3 | 3.1 | 7.3 | 1.8 | 1.4 | 0.2 | 13.1 | 12.9 | 71.2 | 2.2 | 1.8 | 15.0 | 10.3 | 84.4 | 12.7 | 1 |
| Luis Cordero | 61 | 11.0 | 3.2 | 8.9 | 1.6 | 1.3 | 0.3 | 12.4 | 12.7 | 27.8 | 1.6 | 2.3 | 13.3 | 7.8 | 75.6 | 13.1 | 2 |
| Luis Cordero | 62 | 15.3 | 3.3 | 9.2 | 1.2 | 1.4 | 0.2 | 12.7 | 12.7 | 36.2 | 3.3 | 1.8 | 12.0 | 8.3 | 82.1 | 12.4 | 2 |
| Luis Cordero | 63 | 6.8 | 2.0 | 7.6 | 1.0 | 1.4 | 0.2 | 11.5 | 13.0 | 55.4 | 4.7 | 1.3 | 14.5 | 10.8 | 84.9 | 12.9 | 1 |
| Luis Cordero | 64 | 8.5 | 3.5 | 6.9 | 1.2 | 0.9 | 0.2 | 10.7 | 11.5 | 35.6 | 2.5 | 2.4 | 13.5 | 8.8 | 77.8 | 16.1 | 2 |
| Luis Cordero | 65 | 9.0 | 2.6 | 7.9 | 1.5 | 1.6 | 0.3 | 13.6 | 13.1 | 69.6 | 5.4 | 2.9 | 13.8 | 10.5 | 81.8 | 13.4 | 1 |
| Luis Cordero | 66 | 5.8 | 3.0 | 7.2 | 1.5 | 1.8 | 0.4 | 13.5 | 14.0 | 69.8 | 5.1 | 1.7 | 16.3 | 15.5 | 79.3 | 15.6 | 1 |
| Luis Cordero | 67 | 9.5 | 5.2 | 7.3 | 2.2 | 2.1 | 0.4 | 12.8 | 14.6 | 72.8 | 3.9 | 2.8 | 15.8 | 11.0 | 80.3 | 18.1 | 1 |
| Luis Cordero | 68 | 5.8 | 2.5 | 7.5 | 2.1 | 1.8 | 0.4 | 13.0 | 14.1 | 119.8 | 2.8 | 1.7 | 15.5 | 9.3 | 78.3 | 15.2 | 1 |
| Luis Cordero | 69 | 6.8 | 3.4 | 5.3 | 1.2 | 1.2 | 0.2 | 10.9 | 12.3 | 65.4 | 3.4 | 1.3 | 11.5 | 8.0 | 79.4 | 15.4 | 1 |
| Luis Cordero | 70 | 5.0 | 2.8 | 5.8 | 1.5 | 1.5 | 0.3 | 13.3 | 13.1 | 81.6 | 4.4 | 1.2 | 10.0 | 6.3 | 80.0 | 14.6 | 1 |
| Luis Cordero | 71 | 9.0 | 3.5 | 10.0 | 1.9 | 1.9 | 0.2 | 13.6 | 14.4 | 39.0 | 2.3 | 1.6 | 12.0 | 6.5 | 87.3 | 11.7 | 2 |
| Luis Cordero | 72 | 9.8 | 3.0 | 5.3 | 0.8 | 1.2 | 0.2 | 11.8 | 12.3 | 84.6 | 2.7 | 1.4 | 14.5 | 11.5 | 84.1 | 12.7 | 1 |
| Luis Cordero | 73 | 5.3 | 2.1 | 5.1 | 0.9 | 2.5 | 0.5 | 14.3 | 16.0 | 84.8 | 3.1 | 1.3 | 10.0 | 5.5 | 80.6 | 14.4 | 1 |
| Luis Cordero | 74 | 5.3 | 2.7 | 5.6 | 0.7 | 0.7 | 0.1 | 9.8 | 10.8 | 26.0 | 2.2 | 1.5 | 14.0 | 14.0 | 83.0 | 11.5 | 2 |
| Luis Cordero | 75 | 10.8 | 3.1 | 8.3 | 1.3 | 2.1 | 0.4 | 13.8 | 15.0 | 32.2 | 3.4 | 1.7 | 12.0 | 8.0 | 83.3 | 13.4 | 2 |
| Luis Cordero | 76 | 8.0 | 3.1 | 7.8 | 1.3 | 1.4 | 0.2 | 12.7 | 12.8 | 110.6 | 2.3 | 2.1 | 17.8 | 12.3 | 82.3 | 14.4 | 1 |
| Luis Cordero | 77 | 5.0 | 3.5 | 6.5 | 1.4 | 1.5 | 0.3 | 13.1 | 13.2 | 65.4 | 3.6 | 1.5 | 15.0 | 12.0 | 77.7 | 16.6 | 1 |
| Luis Cordero | 78 | 5.3 | 2.5 | 6.9 | 0.8 | 1.2 | 0.3 | 11.3 | 12.5 | 97.8 | 4.2 | 2.0 | 16.5 | 14.3 | 77.5 | 11.5 | 1 |
| Luis Cordero | 79 | 5.5 | 2.8 | 5.6 | 0.7 | 0.7 | 0.1 | 11.3 | 11.1 | 79.6 | 4.9 | 2.0 | 14.8 | 11.5 | 80.1 | 10.8 | 1 |
| Luis Cordero | 80 | 7.5 | 2.8 | 5.4 | 0.9 | 1.7 | 0.2 | 13.4 | 14.1 | 37.2 | 2.5 | 1.5 | 16.0 | 9.5 | 85.8 | 14.7 | 2 |
| Luis Cordero | 81 | 8.0 | 2.5 | 6.1 | 0.9 | 0.7 | 0.2 | 10.4 | 10.6 | 30.6 | 3.6 | 1.7 | 16.8 | 11.5 | 74.5 | 13.7 | 2 |
| Luis Cordero | 82 | 7.3 | 2.4 | 7.2 | 1.2 | 2.1 | 0.4 | 13.8 | 15.0 | 94.6 | 2.4 | 1.7 | 13.5 | 11.3 | 80.2 | 13.9 | 1 |
| Luis Cordero | 83 | 8.5 | 3.5 | 7.6 | 1.0 | 2.4 | 0.4 | 14.0 | 15.3 | 38.0 | 2.5 | 2.0 | 17.3 | 12.8 | 82.7 | 15.7 | 2 |
| Luis Cordero | 84 | 7.0 | 3.4 | 7.0 | 1.5 | 1.5 | 0.4 | 11.9 | 13.7 | 86.6 | 3.8 | 2.1 | 17.8 | 16.3 | 75.6 | 14.4 | 1 |
| Luis Cordero | 85 | 9.0 | 2.8 | 6.9 | 1.2 | 1.9 | 0.3 | 13.1 | 14.1 | 53.0 | 2.8 | 1.4 | 17.5 | 12.8 | 83.3 | 14.4 | 1 |
| Luis Cordero | 86 | 15.5 | 3.6 | 8.9 | 1.1 | 1.5 | 0.3 | 13.0 | 13.6 | 63.6 | 4.5 | 2.3 | 15.5 | 8.8 | 79.0 | 12.5 | 1 |
| Luis Cordero | 87 | 5.5 | 2.6 | 7.1 | 1.4 | 0.7 | 0.1 | 10.3 | 11.6 | 26.8 | 2.1 | 1.3 | 14.3 | 10.0 | 84.0 | 10.4 | 2 |
| Luis Cordero | 88 | 5.5 | 4.2 | 7.6 | 1.5 | 1.2 | 0.3 | 12.3 | 11.6 | 46.6 | 2.8 | 2.2 | 14.3 | 9.8 | 73.3 | 16.1 | 1 |
| Luis Cordero | 89 | 4.5 | 3.5 | 6.8 | 1.2 | 1.6 | 0.3 | 12.6 | 14.8 | 49.4 | 5.0 | 1.5 | 15.0 | 9.8 | 80.7 | 15.0 | 1 |
| Luis Cordero | 90 | 7.0 | 2.5 | 7.8 | 1.1 | 2.3 | 0.4 | 15.1 | 15.4 | 184.0 | 2.4 | 1.7 | 18.0 | 10.8 | 84.7 | 10.7 | 1 |
| Luis Cordero | 91 | 9.3 | 3.2 | 6.2 | 1.0 | 1.5 | 0.4 | 12.0 | 13.8 | 90.6 | 3.7 | 2.0 | 16.3 | 11.0 | 75.1 | 14.8 | 1 |
| Luis Cordero | 92 | 6.0 | 2.6 | 5.7 | 1.0 | 0.6 | 0.1 | 10.5 | 10.3 | 77.2 | 2.4 | 1.5 | 18.8 | 10.0 | 75.8 | 9.8 | 1 |
| Luis Cordero | 93 | 6.5 | 2.7 | 7.1 | 1.1 | 1.4 | 0.3 | 12.5 | 13.1 | 68.0 | 2.7 | 1.4 | 13.5 | 9.8 | 81.8 | 18.4 | 1 |
| Luis Cordero | 94 | 5.8 | 2.2 | 6.2 | 1.2 | 1.0 | 0.2 | 11.1 | 11.6 | 8.8 | 2.3 | 1.5 | 15.3 | 8.0 | 81.2 | 12.4 | 2 |
| Luis Cordero | 95 | 10.8 | 3.3 | 8.2 | 1.4 | 1.1 | 0.2 | 11.7 | 12.0 | 62.0 | 1.9 | 1.6 | 15.8 | 10.8 | 82.8 | 11.5 | 1 |
| Luis Cordero | 96 | 8.0 | 2.9 | 8.0 | 1.6 | 1.9 | 0.3 | 13.5 | 14.3 | 34.6 | 3.4 | 1.7 | 14.0 | 6.5 | 83.5 | 15.8 | 2 |
| Luis Cordero | 97 | 8.0 | 3.9 | 9.4 | 1.4 | 1.9 | 0.3 | 14.4 | 14.6 | 28.6 | 2.6 | 2.1 | 16.5 | 11.8 | 83.9 | 13.8 | 2 |
| Luis Cordero | 98 | 8.0 | 2.4 | 7.7 | 1.3 | 1.9 | 0.3 | 14.6 | 14.4 | 13.2 | 2.5 | 1.6 | 18.5 | 8.5 | 85.2 | 11.8 | 2 |
| Luis Cordero | 99 | 7.3 | 2.0 | 6.6 | 1.3 | 2.0 | 0.3 | 14.2 | 14.5 | 50.6 | 1.9 | 1.5 | 15.5 | 8.0 | 86.0 | 11.9 | 1 |
| Luis Cordero | 100 | 8.0 | 4.3 | 9.1 | 1.2 | 2.3 | 0.4 | 14.3 | 15.7 | 36.0 | 1.2 | 2.4 | 11.5 | 7.3 | 82.6 | 14.6 | 2 |
| San Vicente | 101 | 4.3 | 3.1 | 7.1 | 1.5 | 1.1 | 0.2 | 11.5 | 11.8 | 26.4 | 2.4 | 2.1 | 12.5 | 9.3 | 82.7 | 18.2 | 2 |
| San Vicente | 102 | 3.8 | 3.4 | 6.8 | 1.4 | 1.1 | 0.2 | 13.2 | 13.4 | 39.2 | 2.2 | 2.8 | 12.5 | 8.5 | 80.1 | 14.8 | 1 |
| San Vicente | 103 | 4.8 | 2.2 | 6.0 | 1.8 | 1.5 | 0.3 | 12.5 | 14.5 | 30.6 | 2.0 | 2.0 | 14.0 | 10.8 | 83.1 | 11.8 | 2 |
| San Vicente | 104 | 4.5 | 3.7 | 9.9 | 1.5 | 1.3 | 0.2 | 12.6 | 12.7 | 12.4 | 2.5 | 1.9 | 11.8 | 9.8 | 85.3 | 17.4 | 2 |
| San Vicente | 105 | 5.0 | 1.8 | 6.0 | 1.1 | 1.7 | 0.3 | 13.9 | 13.9 | 19.8 | 2.3 | 2.1 | 12.3 | 8.5 | 83.6 | 14.0 | 2 |
| San Vicente | 106 | 5.0 | 2.9 | 7.8 | 1.2 | 1.1 | 0.2 | 11.0 | 12.3 | 63.0 | 2.9 | 1.7 | 7.8 | 8.3 | 79.8 | 20.4 | 1 |
| San Vicente | 107 | 5.3 | 3.0 | 7.7 | 1.2 | 0.7 | 0.1 | 10.3 | 11.0 | 21.8 | 3.3 | 1.4 | 11.3 | 10.8 | 86.6 | 8.8 | 2 |
| San Vicente | 108 | 4.0 | 3.3 | 7.6 | 0.8 | 1.4 | 0.2 | 11.6 | 14.6 | 30.6 | 1.8 | 1.9 | 14.3 | 11.8 | 86.8 | 8.7 | 2 |
| San Vicente | 109 | 5.8 | 4.2 | 8.8 | 1.8 | 0.9 | 0.1 | 10.8 | 27.4 | 74.2 | 2.1 | 2.2 | 15.8 | 15.0 | 84.5 | 8.0 | 1 |
| San Vicente | 110 | 7.3 | 3.5 | 8.1 | 1.2 | 1.2 | 0.2 | 12.8 | 12.8 | 65.8 | 3.5 | 2.2 | 11.3 | 10.5 | 84.4 | 9.9 | 1 |
| San Vicente | 111 | 6.0 | 2.9 | 6.7 | 1.8 | 1.1 | 0.2 | 12.3 | 12.7 | 30.0 | 2.4 | 1.5 | 14.0 | 11.5 | 78.4 | 14.2 | 2 |
| San Vicente | 112 | 6.3 | 3.4 | 6.6 | 0.8 | 2.4 | 0.6 | 14.9 | 16.0 | 15.2 | 1.8 | 1.8 | 14.0 | 9.5 | 76.5 | 15.9 | 2 |
| San Vicente | 113 | 7.0 | 2.9 | 8.6 | 1.5 | 1.1 | 0.2 | 11.3 | 11.9 | 30.0 | 1.8 | 1.6 | 15.3 | 14.8 | 80.9 | 18.0 | 2 |
| San Vicente | 114 | 6.8 | 4.2 | 7.6 | 1.3 | 1.9 | 0.4 | 13.8 | 15.6 | 18.6 | 2.2 | 1.6 | 10.3 | 9.3 | 77.6 | 10.0 | 2 |
| San Vicente | 115 | 5.5 | 2.4 | 7.0 | 1.2 | 2.1 | 0.4 | 15.4 | 14.8 | 37.2 | 2.1 | 1.4 | 15.3 | 7.8 | 80.5 | 18.0 | 2 |
| San Vicente | 116 | 6.0 | 2.9 | 7.4 | 0.8 | 2.0 | 6.9 | 15.1 | 14.5 | 21.0 | 1.4 | 1.9 | 11.0 | 9.3 | 81.3 | 17.4 | 2 |
| San Vicente | 117 | 3.3 | 3.2 | 7.1 | 0.9 | 1.8 | 0.3 | 15.0 | 14.0 | 45.0 | 1.4 | 0.9 | 11.0 | 9.0 | 81.2 | 16.2 | 1 |
| San Vicente | 118 | 5.0 | 3.7 | 8.8 | 1.9 | 1.7 | 0.3 | 13.1 | 15.2 | 20.4 | 1.9 | 1.9 | 13.0 | 12.5 | 82.8 | 12.1 | 2 |
| San Vicente | 119 | 4.5 | 2.8 | 6.3 | 1.7 | 1.2 | 0.2 | 12.2 | 13.0 | 8.2 | 2.1 | 1.0 | 12.5 | 11.5 | 81.2 | 13.1 | 2 |
| San Vicente | 120 | 5.5 | 3.2 | 7.6 | 1.0 | 1.4 | 0.3 | 12.5 | 13.0 | 32.6 | 1.9 | 1.0 | 12.8 | 11.0 | 81.3 | 22.0 | 2 |
| San Vicente | 121 | 5.0 | 3.1 | 7.2 | 1.5 | 0.9 | 0.2 | 11.3 | 11.2 | 18.0 | 1.8 | 1.5 | 16.0 | 11.8 | 82.0 | 15.0 | 2 |
| San Vicente | 122 | 3.0 | 3.0 | 6.3 | 1.3 | 0.7 | 0.1 | 10.4 | 10.3 | 23.4 | 2.2 | 1.7 | 15.3 | 10.0 | 82.1 | 17.6 | 2 |
| San Vicente | 123 | 6.8 | 2.7 | 7.3 | 1.4 | 0.9 | 0.2 | 11.2 | 11.8 | 57.4 | 2.2 | 2.4 | 18.0 | 16.8 | 75.6 | 13.5 | 1 |
| San Vicente | 124 | 6.0 | 3.6 | 7.6 | 1.0 | 1.2 | 0.2 | 12.4 | 13.3 | 26.6 | 1.7 | 1.9 | 16.8 | 13.8 | 85.6 | 6.1 | 2 |
| San Vicente | 125 | 3.5 | 3.3 | 6.6 | 0.8 | 1.2 | 0.2 | 12.3 | 13.3 | 9.0 | 1.8 | 1.5 | 10.5 | 9.0 | 84.1 | 11.3 | 2 |
| San Vicente | 126 | 6.3 | 3.1 | 8.0 | 0.9 | 1.1 | 0.2 | 12.0 | 13.0 | 14.0 | 1.9 | 1.8 | 12.5 | 13.5 | 81.4 | 16.0 | 2 |
| San Vicente | 127 | 9.8 | 3.7 | 9.1 | 1.4 | 2.3 | 0.4 | 16.3 | 15.6 | 54.8 | 2.4 | 2.4 | 15.5 | 13.3 | 81.6 | 11.2 | 1 |
| San Vicente | 128 | 6.0 | 1.8 | 6.1 | 1.4 | 0.8 | 0.2 | 11.7 | 11.4 | 73.0 | 3.3 | 2.1 | 14.0 | 11.0 | 78.9 | 16.2 | 1 |
| San Vicente | 129 | 4.5 | 3.0 | 6.4 | 0.7 | 1.4 | 0.2 | 12.5 | 12.9 | 12.8 | 1.5 | 0.9 | 13.0 | 8.3 | 85.7 | 19.0 | 2 |
| San Vicente | 130 | 5.5 | 3.1 | 5.7 | 1.1 | 1.9 | 0.3 | 13.9 | 14.9 | 35.8 | 2.8 | 1.5 | 14.3 | 9.8 | 85.0 | 15.6 | 2 |
| San Vicente | 131 | 3.8 | 2.4 | 6.2 | 1.0 | 1.5 | 0.3 | 12.4 | 14.0 | 74.8 | 2.9 | 1.5 | 15.3 | 11.3 | 82.8 | 17.2 | 1 |
| San Vicente | 132 | 7.5 | 4.0 | 9.9 | 2.1 | 2.1 | 0.4 | 14.5 | 15.4 | 14.0 | 2.8 | 1.8 | 16.0 | 11.5 | 81.7 | 17.6 | 2 |
| San Vicente | 133 | 5.8 | 2.7 | 7.0 | 0.8 | 1.0 | 0.2 | 13.5 | 12.1 | 32.4 | 1.3 | 1.1 | 12.3 | 9.5 | 79.2 | 16.6 | 2 |
| San Vicente | 134 | 5.0 | 3.1 | 6.6 | 1.1 | 0.9 | 0.2 | 9.9 | 11.9 | 23.6 | 2.0 | 1.6 | 15.0 | 10.8 | 81.7 | 19.6 | 2 |
| San Vicente | 135 | 6.3 | 3.0 | 6.9 | 1.1 | 1.6 | 0.3 | 13.5 | 13.3 | 46.2 | 2.3 | 2.0 | 13.0 | 8.3 | 80.8 | 19.0 | 1 |
| San Vicente | 136 | 9.3 | 3.4 | 8.6 | 0.8 | 1.6 | 0.3 | 12.5 | 14.1 | 38.2 | 2.1 | 2.4 | 13.0 | 9.8 | 80.4 | 18.4 | 2 |
| San Vicente | 137 | 6.8 | 2.3 | 7.2 | 1.3 | 1.5 | 0.2 | 13.9 | 13.6 | 15.4 | 2.1 | 2.0 | 12.0 | 9.0 | 86.6 | 14.2 | 2 |
| San Vicente | 138 | 7.8 | 3.3 | 8.1 | 0.7 | 1.3 | 0.2 | 11.5 | 11.9 | 33.6 | 2.4 | 1.6 | 12.0 | 9.0 | 84.7 | 13.4 | 2 |
| San Vicente | 139 | 6.0 | 2.8 | 8.2 | 1.1 | 1.0 | 0.2 | 12.9 | 12.1 | 40.4 | 2.5 | 1.3 | 13.0 | 12.0 | 79.6 | 15.6 | 1 |
| San Vicente | 140 | 6.0 | 4.2 | 8.2 | 1.0 | 1.3 | 0.2 | 12.3 | 12.5 | 27.4 | 1.6 | 1.2 | 17.0 | 7.0 | 82.3 | 20.8 | 2 |
| San Vicente | 141 | 9.8 | 3.4 | 8.9 | 1.3 | 2.1 | 0.4 | 13.7 | 15.0 | 37.2 | 2.3 | 1.6 | 15.3 | 13.3 | 83.0 | 17.2 | 2 |
| San Vicente | 142 | 2.3 | 2.6 | 5.3 | 0.9 | 2.2 | 0.4 | 14.5 | 15.3 | 17.0 | 2.4 | 1.4 | 13.0 | 9.8 | 81.9 | 21.8 | 2 |
| San Vicente | 143 | 6.5 | 4.0 | 7.7 | 1.4 | 2.4 | 0.4 | 16.7 | 15.7 | 35.4 | 3.2 | 3.3 | 16.5 | 10.3 | 83.0 | 16.6 | 2 |
| San Vicente | 144 | 9.0 | 3.4 | 7.9 | 1.0 | 1.8 | 0.3 | 13.9 | 14.5 | 49.8 | 2.9 | 2.0 | 13.0 | 7.3 | 82.3 | 17.6 | 1 |
| San Vicente | 145 | 4.0 | 3.4 | 7.3 | 0.8 | 1.5 | 0.2 | 13.8 | 13.1 | 10.4 | 2.0 | 1.9 | 13.3 | 11.3 | 84.1 | 14.4 | 2 |
| San Vicente | 146 | 7.0 | 2.6 | 5.3 | 0.9 | 0.9 | 0.2 | 11.3 | 11.8 | 47.8 | 1.9 | 1.1 | 15.5 | 13.3 | 76.8 | 16.2 | 1 |
| San Vicente | 147 | 7.0 | 2.4 | 8.4 | 1.4 | 1.0 | 0.3 | 12.1 | 12.5 | 49.4 | 1.9 | 1.3 | 15.0 | 12.8 | 74.9 | 23.2 | 1 |
| San Vicente | 148 | 6.3 | 3.5 | 9.0 | 1.3 | 1.2 | 0.2 | 12.0 | 13.2 | 25.2 | 2.1 | 1.6 | 16.0 | 7.8 | 84.4 | 8.5 | 2 |
| San Vicente | 149 | 4.5 | 4.8 | 7.3 | 1.5 | 1.6 | 0.3 | 13.4 | 13.9 | 44.4 | 1.9 | 2.4 | 14.8 | 10.0 | 83.4 | 12.4 | 1 |
| San Vicente | 150 | 11.5 | 2.5 | 8.9 | 1.1 | 1.4 | 0.3 | 13.0 | 14.0 | 40.0 | 1.6 | 1.8 | 17.0 | 11.5 | 81.4 | 12.2 | 2 |
| Nabon | 151 | 4.1 | 2.6 | 4.8 | 2.9 | 2.2 | 0.6 | 15.0 | 14.0 | 41.3 | 3.8 | 3.3 | 17.0 | 20.0 | 72.7 | 17.1 | 1 |
| Nabon | 152 | 5.0 | 1.6 | 4.9 | 3.2 | 1.6 | 0.3 | 13.8 | 13.8 | 113.4 | 3.7 | 4.1 | 12.0 | 19.0 | 80.9 | 30.4 | 1 |
| Nabon | 153 | 5.0 | 1.8 | 3.5 | 3.7 | 1.2 | 0.2 | 13.3 | 13.7 | 18.0 | 2.9 | 2.9 | 20.5 | 16.0 | 81.7 | 12.1 | 2 |
| Nabon | 154 | 8.2 | 2.6 | 5.6 | 3.5 | 1.3 | 0.2 | 14.5 | 13.0 | 24.0 | 3.3 | 3.9 | 20.7 | 28.0 | 81.1 | 16.0 | 2 |
| Nabon | 155 | 6.2 | 2.5 | 5.6 | 3.3 | 1.4 | 0.3 | 13.7 | 13.2 | 23.3 | 4.4 | 3.8 | 19.3 | 11.5 | 80.9 | 12.4 | 2 |
| Nabon | 156 | 7.2 | 2.6 | 4.6 | 3.7 | 1.1 | 0.2 | 11.5 | 12.2 | 27.3 | 2.8 | 3.1 | 20.3 | 22.0 | 82.5 | 15.1 | 2 |
| Nabon | 157 | 7.0 | 1.9 | 5.0 | 3.5 | 1.3 | 0.2 | 13.8 | 13.6 | 12.5 | 3.8 | 3.4 | 11.0 | 9.5 | 83.2 | 8.1 | 2 |
| Nabon | 158 | 6.0 | 1.6 | 4.4 | 3.1 | 1.2 | 0.2 | 12.5 | 12.8 | 13.0 | 4.4 | 4.0 | 11.3 | 13.5 | 80.9 | 9.2 | 2 |
| Nabon | 159 | 7.2 | 1.9 | 5.0 | 4.2 | 1.4 | 0.3 | 13.5 | 14.0 | 29.8 | 1.9 | 3.0 | 14.0 | 12.0 | 82.4 | 12.1 | 2 |
| Nabon | 160 | 7.6 | 2.5 | 5.7 | 4.9 | 1.9 | 0.3 | 14.8 | 14.0 | 15.8 | 2.2 | 3.1 | 13.8 | 11.8 | 81.3 | 9.3 | 2 |
| Nabon | 161 | 6.0 | 2.0 | 4.4 | 4.3 | 1.9 | 0.3 | 14.2 | 14.8 | 24.7 | 2.9 | 3.2 | 14.5 | 19.5 | 84.6 | 12.9 | 2 |
| Nabon | 162 | 11.4 | 2.7 | 5.9 | 3.3 | 1.1 | 0.2 | 12.6 | 11.9 | 12.0 | 3.4 | 3.3 | 15.0 | 11.8 | 85.2 | 9.1 | 2 |
| Nabon | 163 | 6.4 | 2.9 | 5.8 | 3.6 | 1.5 | 0.2 | 11.0 | 13.8 | 20.8 | 1.7 | 3.0 | 15.0 | 9.0 | 84.6 | 9.9 | 2 |
| Nabon | 164 | 6.2 | 1.5 | 3.9 | 3.6 | 1.1 | 0.2 | 13.1 | 11.9 | 20.0 | 2.6 | 3.4 | 13.3 | 8.8 | 85.5 | 9.6 | 2 |
| Nabon | 165 | 10.0 | 2.3 | 8.1 | 4.5 | 2.1 | 0.4 | 16.8 | 14.8 | 36.8 | 3.1 | 2.9 | 11.3 | 11.5 | 82.9 | 13.1 | 2 |
| Nabon | 166 | 6.6 | 3.2 | 5.9 | 3.6 | 2.2 | 0.4 | 13.5 | 15.4 | 58.2 | 2.2 | 2.7 | 7.0 | 10.7 | 80.1 | 16.2 | 1 |
| Nabon | 167 | 5.4 | 2.0 | 5.3 | 3.3 | 2.0 | 0.4 | 14.4 | 14.9 | 13.7 | 3.7 | 3.3 | 9.5 | 10.3 | 80.7 | 8.1 | 2 |
| Nabon | 168 | 5.6 | 2.7 | 4.3 | 4.2 | 1.6 | 0.3 | 13.2 | 12.6 | 40.6 | 3.7 | 3.6 | 14.7 | 11.3 | 79.6 | 14.8 | 1 |
| Nabon | 169 | 6.8 | 2.6 | 5.7 | 3.8 | 1.5 | 0.3 | 11.9 | 12.9 | 78.4 | 3.2 | 3.8 | 10.8 | 10.3 | 79.4 | 21.3 | 1 |
| Nabon | 170 | 6.0 | 2.4 | 5.1 | 3.5 | 1.7 | 0.3 | 12.5 | 13.7 | 9.8 | 2.3 | 2.8 | 14.7 | 13.3 | 80.0 | 8.6 | 2 |
| Nabon | 171 | 7.0 | 1.8 | 5.6 | 4.2 | 1.9 | 0.4 | 13.3 | 13.9 | 54.7 | 3.3 | 3.0 | 20.0 | 14.8 | 80.3 | 19.1 | 1 |
| Nabon | 172 | 8.2 | 2.3 | 5.7 | 7.0 | 2.2 | 0.3 | 15.8 | 15.5 | 3.0 | 1.8 | 2.9 | 14.5 | 8.5 | 85.7 | 6.1 | 2 |
| Nabon | 173 | 6.6 | 2.0 | 4.0 | 5.2 | 2.4 | 0.3 | 15.2 | 15.4 | 18.5 | 3.8 | 3.4 | 16.0 | 5.8 | 87.2 | 9.5 | 2 |
| Nabon | 174 | 5.0 | 2.3 | 3.7 | 3.5 | 3.1 | 0.6 | 15.0 | 16.5 | 14.8 | 3.4 | 3.7 | 14.0 | 11.3 | 80.6 | 9.4 | 2 |
| Nabon | 175 | 6.2 | 2.7 | 7.2 | 4.3 | 1.9 | 0.2 | 12.8 | 14.2 | 13.0 | 2.2 | 3.2 | 16.0 | 15.0 | 86.8 | 9.9 | 2 |
| Nabon | 176 | 10.0 | 3.9 | 7.5 | 4.5 | 2.5 | 0.4 | 14.8 | 16.2 | 29.2 | 2.9 | 2.5 | 9.3 | 8.3 | 82.8 | 10.4 | 2 |
| Nabon | 177 | 7.5 | 3.0 | 6.5 | 4.3 | 2.0 | 0.4 | 14.4 | 14.9 | 22.8 | 4.6 | 3.3 | 10.5 | 7.5 | 80.5 | 9.7 | 2 |
| Nabon | 178 | 9.5 | 3.1 | 7.3 | 3.6 | 1.4 | 0.2 | 11.9 | 13.4 | 36.2 | 2.7 | 2.5 | 9.0 | 8.8 | 85.7 | 11.8 | 2 |
| Nabon | 179 | 5.5 | 2.7 | 5.5 | 4.6 | 1.6 | 0.3 | 13.4 | 14.1 | 18.8 | 3.7 | 2.3 | 12.3 | 9.8 | 84.0 | 10.8 | 2 |
| Nabon | 180 | 5.5 | 3.3 | 6.1 | 3.7 | 2.7 | 0.4 | 15.3 | 17.0 | 30.8 | 5.0 | 2.3 | 14.8 | 7.3 | 83.9 | 12.0 | 2 |
| Nabon | 181 | 7.8 | 3.2 | 8.3 | 3.5 | 2.9 | 0.4 | 13.8 | 13.5 | 55.4 | 2.7 | 2.2 | 11.5 | 10.0 | 87.7 | 16.4 | 1 |
| Nabon | 182 | 7.3 | 2.5 | 7.6 | 3.4 | 2.8 | 0.5 | 15.8 | 16.3 | 23.2 | 2.7 | 2.7 | 13.0 | 8.0 | 81.5 | 9.9 | 2 |
| Nabon | 183 | 8.8 | 2.7 | 7.1 | 2.6 | 2.8 | 0.5 | 15.3 | 16.9 | 24.0 | 3.2 | 2.8 | 7.8 | 5.5 | 80.6 | 8.7 | 2 |
| Nabon | 184 | 8.0 | 2.4 | 6.5 | 3.4 | 2.6 | 0.4 | 15.1 | 16.5 | 43.6 | 2.1 | 2.9 | 13.0 | 7.8 | 81.4 | 13.9 | 2 |
| Nabon | 185 | 6.8 | 3.5 | 8.3 | 4.0 | 3.6 | 0.3 | 17.3 | 18.2 | 28.0 | 2.0 | 2.4 | 11.5 | 6.8 | 81.3 | 10.1 | 2 |
| Nabon | 186 | 6.0 | 2.3 | 5.7 | 3.4 | 3.6 | 0.7 | 16.8 | 18.2 | 7.6 | 1.9 | 2.4 | 14.3 | 7.8 | 81.7 | 6.8 | 2 |
| Nabon | 187 | 9.8 | 2.8 | 6.3 | 3.6 | 3.1 | 0.6 | 16.1 | 17.1 | 25.4 | 2.9 | 2.1 | 8.5 | 7.3 | 80.7 | 9.2 | 2 |
| Nabon | 188 | 5.5 | 3.2 | 6.9 | 3.6 | 2.0 | 0.3 | 14.0 | 15.0 | 25.6 | 2.4 | 2.2 | 15.0 | 9.3 | 83.8 | 10.9 | 2 |
| Nabon | 189 | 6.8 | 2.9 | 6.3 | 3.6 | 1.5 | 0.3 | 13.0 | 13.6 | 13.6 | 2.2 | 2.4 | 9.3 | 7.5 | 83.6 | 7.0 | 2 |
| Nabon | 190 | 7.3 | 2.6 | 6.6 | 3.6 | 1.7 | 0.3 | 13.8 | 14.2 | 28.6 | 2.0 | 1.8 | 13.0 | 10.5 | 84.9 | 11.2 | 2 |
| Nabon | 191 | 6.3 | 2.7 | 8.6 | 3.3 | 1.8 | 0.3 | 13.8 | 14.2 | 15.2 | 3.4 | 3.2 | 21.3 | 6.3 | 84.4 | 9.9 | 2 |
| Nabon | 192 | 4.8 | 2.6 | 5.7 | 2.9 | 1.7 | 0.3 | 12.6 | 14.6 | 34.2 | 2.8 | 2.3 | 13.0 | 8.8 | 84.2 | 12.2 | 2 |
| Nabon | 193 | 9.8 | 3.2 | 8.8 | 2.8 | 1.3 | 0.2 | 11.8 | 13.0 | 38.6 | 4.2 | 3.0 | 10.0 | 8.0 | 83.5 | 12.8 | 2 |
| Nabon | 194 | 4.8 | 2.7 | 5.4 | 2.8 | 1.6 | 0.3 | 12.5 | 13.8 | 44.6 | 2.2 | 1.5 | 15.0 | 6.8 | 82.6 | 14.0 | 1 |
| Nabon | 195 | 6.8 | 2.2 | 8.1 | 3.8 | 1.7 | 0.3 | 12.6 | 12.1 | 30.6 | 2.5 | 2.7 | 17.8 | 8.3 | 83.5 | 12.4 | 2 |
| Nabon | 196 | 7.0 | 2.4 | 5.9 | 3.4 | 2.8 | 0.5 | 16.2 | 16.5 | 15.2 | 2.7 | 2.5 | 15.8 | 9.8 | 82.3 | 9.2 | 2 |
| Nabon | 197 | 6.3 | 2.9 | 5.4 | 2.9 | 1.7 | 0.6 | 15.3 | 16.7 | 35.4 | 1.8 | 1.9 | 10.0 | 8.0 | 66.0 | 11.4 | 2 |
| Nabon | 198 | 5.3 | 2.3 | 5.5 | 2.5 | 2.0 | 0.3 | 13.0 | 14.9 | 32.8 | 1.5 | 2.4 | 11.0 | 8.5 | 83.5 | 11.2 | 2 |
| Nabon | 199 | 3.5 | 2.2 | 5.3 | 3.8 | 1.6 | 0.3 | 12.5 | 14.4 | 30.4 | 1.9 | 2.2 | 13.0 | 10.0 | 83.7 | 11.5 | 2 |
| Nabon | 200 | 5.3 | 1.7 | 6.2 | 3.0 | 1.7 | 0.3 | 12.6 | 13.9 | 26.4 | 4.4 | 2.7 | 17.3 | 11.3 | 84.5 | 12.4 | 2 |

**Supplementary Table 3**. Descriptive statistics of morphological parameters (SNF, H, FWD, FL, P) for the entire sample and separated by morphological groups 1 and 2, including mean, minimum, maximum, standard deviation (SD), coefficient of variation (CV), and sample size (n).

| **Parameter** | **SNF** | **H** | **FWD** | **FL** | **P** |
| --- | --- | --- | --- | --- | --- |
| Media | 49.03 | 82.08 | 13.81 | 12.97 | 6.44 |
| Min | 3 | 66.02 | 10.27 | 9.04 | 2.25 |
| Max | 188.8 | 93.09 | 27.37 | 17.31 | 15.5 |
| SD_all | 33.21 | 3.26 | 1.78 | 1.5 | 2.07 |
| CV_all | 67.74 | 3.97 | 12.9 | 11.56 | 32.22 |
| n_all | 200 | 200 | 200 | 200 | 200 |
| Media morphological group 1 | 89.01 | 81.39 | 13.77 | 12.61 | 6.22 |
| SD morphological group 1 | 28.51 | 3.45 | 2.24 | 1.22 | 2.26 |
| CV morphological group 1 | 32.03 | 4.24 | 16.28 | 9.65 | 36.33 |
| n morphological group 1 | 62 | 62 | 62 | 62 | 62 |
| Media morphological group 2 | 31.06 | 82.39 | 13.83 | 13.13 | 6.53 |
| SD morphological group 2 | 13.83 | 3.14 | 1.54 | 1.59 | 1.99 |
| CV morphological group 2 | 44.53 | 3.81 | 11.14 | 12.09 | 30.4 |
| n morphological group 2 | 138 | 138 | 138 | 138 | 138 |
